# Supplementary figures and images for: A spider mating plug functions to protect sperm
Source: PLoS One. 2024 Mar 29;19(3):e0301290. doi: 10.1371/journal.pone.0301290 (PMC10980215; doi:10.1371/journal.pone.0301290)

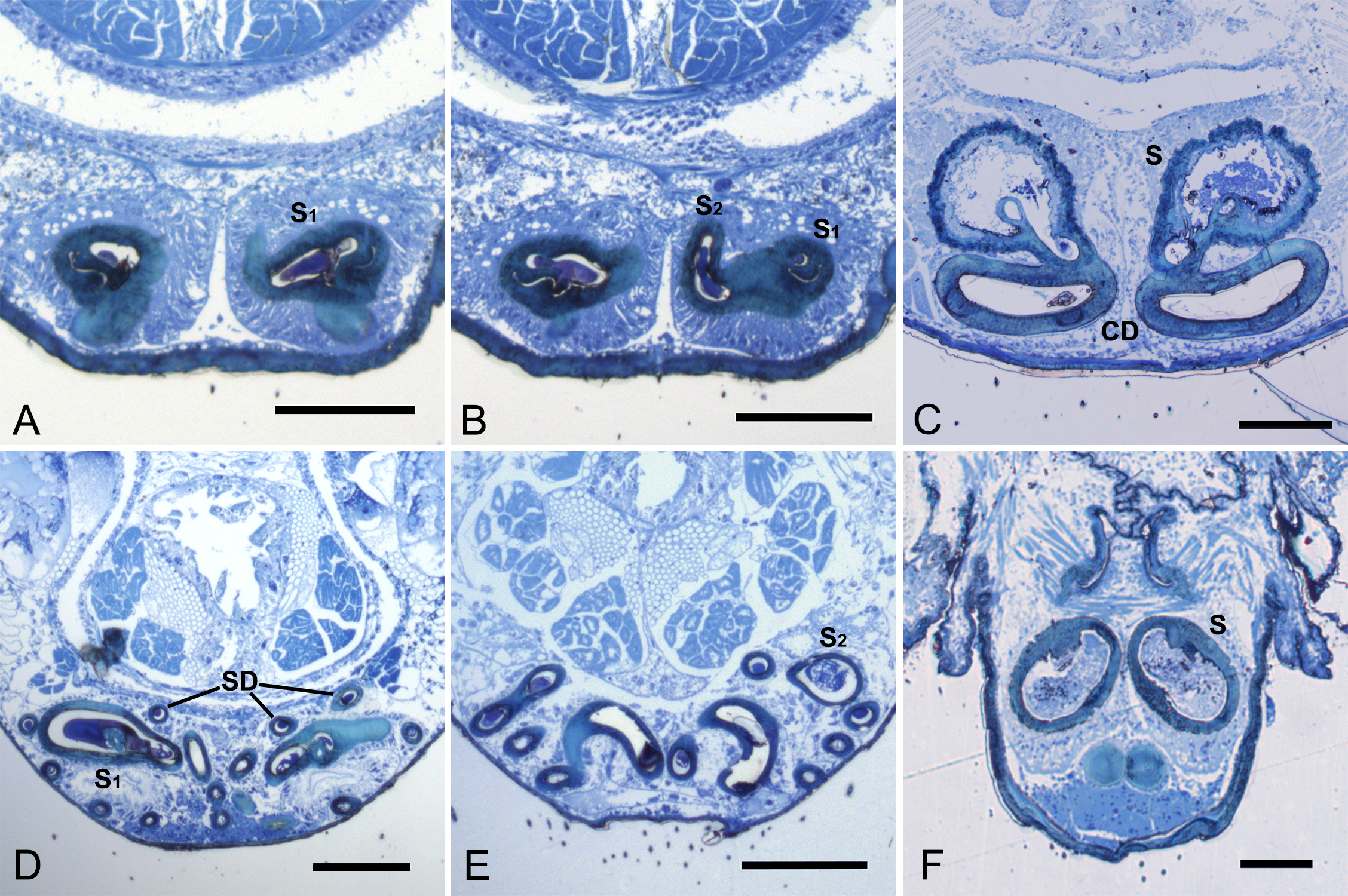

Supplement: S1 Fig — A−B, Neriene emphana (Linyphiidae); C, Parasteatoda tepidariorum (Theridiidae); D−E, Hahnia zhejiangensis (Hahniidae); F, Neoscona sp. (Araneidae). CD, copulatory duct; S1, primary spermatheca; S2, secondary spermatheca; SD, spermathecal duct. Scale bars: 0.1 mm. (TIF) [file pone.0301290.s001.tif]
