# Supplementary material for: A spider mating plug functions to protect sperm
Source: PLoS One. 2024 Mar 29;19(3):e0301290. doi: 10.1371/journal.pone.0301290 (PMC10980215; doi:10.1371/journal.pone.0301290)
Supplement: S1 Table — (DOCX) [file pone.0301290.s002.docx]

**S1 Table.** Collecting data of materials examined in this study

| **Number** | **Family name** | **Species name** | **Collecting locality** |
| --- | --- | --- | --- |
| No. 001 | Agelenidae | *Agelena silvatica* | China: Guizhou Province, Kaiyang County, Nanjiang Canyon, N26.95926º: E106.76392º, alt. 904m.. |
| No. 002 | Agelenidae | *Agelena silvatica* | China: Henan Province, Dengfeng City, Shaolin Temple. |
| No. 003 | Agelenidae | *Agelena* sp. | China: Heilongjiang Province, Harbin City, Northeast Forestry University Arboretum. |
| No. 004 | Agelenidae | *Agelena* sp. | China: Heilongjiang Province, Harbin，Northeast Forestry University. |
| No. 005 | Agelenidae | *Agelena* sp. | China: Heilongjiang Province, Harbin，Northeast Forestry University. |
| No. 006 | Agelenidae | Coelotinae sp. | China: Xinjiang Autonomous Region, Yili City, Fruit Ditch, N44.45337º: E81.17210º, alt. 1894m. |
| No. 007 | Agelenidae | *Iwogumoa dicranata* | China: Beijing City, Mt. Yangtai, Jinshan Temple, N40.06750º: E116.08110º, alt. 372m. |
| No. 008 | Agelenidae | *Iwogumoa dicranata* | China: Beijing City, Mt. Songshan, Haituo. |
| No. 009 | Agelenidae | *Pireneitega taishanensis* | China: Beijing City, Mt. Songshan, Haituo. |
| No. 010 | Agelenidae | *Tamgrinia alveolifera* | China: Tibet Autonomous Region, Yadong County. |
| No. 012 | Agelenidae | *Tamgrinia* sp.2 | China: Beijing City, Mt. Songshan, Haituo. |
| No. 011 | Agelenidae | *Tamgrinia* sp2. | China: Beijing City, Mt. Songshan, Haituo. |
| No. 019 | Araneidae | *Aculepeira* sp. | China: Tibet, Shannan, Konggar County, N29.29619º: E91.06770º, alt. 3568m. |
| No. 018 | Araneidae | *Aculepeira* sp. | China: Tibet, Shannan, Konggar County, N29.29619º: E91.06770º, alt. 3568m. |
| No. 021 | Araneidae | *Araneus diadematoides* | China: Beijing City, Mt. Songshan, Haituo. |
| No. 020 | Araneidae | *Araneus diadematoides* | China: Beijing City, Mt. Yangtai, Jinshan Temple, N40.0675º: E116.0811º, alt. 372m. |
| No. 023 | Araneidae | *Araneus* sp. | China: Jiangxi Province, Mt. Jinggangshan, Fairy Fall, N26.58415º: E114.13800º, alt. 600-800m. |
| No. 024 | Araneidae | *Araneus ventricosus* | China: Heilongjiang Province, Wudalianchi Scenic Area, N48.65191º: E126.16434º, alt. 377m. |
| No. 026 | Araneidae | *Argiope minuta* | China: Guizhou Province, Anshun County, Huangguoshu National Park, N25.98668º: E105.65593º. |
| No. 030 | Araneidae | *Cyclosa* sp. | China: Jiangxi Province, Xiannv Pond, N26.58415º: E114.13797º, alt. 600-800. |
| No. 031 | Araneidae | *Cyclosa* sp. | China: Guizhou Province, Xifeng Concentration Camp, N27.03669º: E106.73502º, alt. 890m. |
| No. 034 | Araneidae | *Gasteracantha* sp. | China: Guizhou Province, Anshun County, Longgong Caves, N26.09858º: E106.87989º, alt. 1195m. |
| No. 036 | Araneidae | *Hypsosinga sanguinea* | China: Beijing City, Mt. Songshan, the way to Dazhuangke. |
| No. 037 | Araneidae | *Hypsosinga* sp.2 | China: Guizhou Province, Tianlong Tunpu, Mt. Tiantai, N26.34566º: E106.17463º, alt. 1398m. |
| No. 044 | Araneidae | *Neoscona* sp.1 | China: Beijing City, Mt. Yangtai, Jinshan Temple, N40.0675º: E116.0811º, alt. 372m,. |
| No. 045 | Araneidae | *Neoscona* sp.1 | China: Beijing City, Mt. Yangtai, Jinshan Temple, N40.0675º: E116.0811º, alt. 372m,. |
| No. 046 | Araneidae | *Neoscona* sp.2 | China: Guizhou Province, Zunyi City, Mt. Red Army, N27.68907º: E106.90571º. |
| No. 053 | Clubionidae | *Clubiona duoconcava* | China: Beijing City, Mt. Songshan, Haituo. |
| No. 054 | Clubionidae | *Clubiona flexa* | China: Beijing City, Mt. Songshan, Haituo. |
| No. 055 | Dictynidae | *Lathys* sp. | China: Beijing City, Mt. Yangtai, Dajue Temple, N40.05115º: E116.10132º, alt. 219m. |
| No. 056 | Dictynidae | *Lathys stigmatisata* | China: Xinjiang Autonomous Region, Yili City, Fruit Ditch, N44.45337º: E81.17210º, alt. 1894m. |
| No. 057 | Dictynidae | *Lathys stigmatisata* | China: Xinjiang Autonomous Region, Yili City, Fruit Ditch, N44.45337º: E81.17210º, alt. 1894m. |
| No. 058 | Dictynidae | *Lathys stigmatisata* | China: Xinjiang Autonomous Region, Yili City, Fruit Ditch, N44.45337º: E81.17210º, alt. 1894m. |
| No. 066 | Gnaphosidae | *Gnaphosa kompirensis* | China: Beijing City, Mt. Songshan, Haituo. |
| No. 067 | Gnaphosidae | *Gnaphosa moerens* | China: Beijing City, Mt. Songshan, Haituo. |
| No. 068 | Gnaphosidae | *Gnaphosa* sp. | China: Beijing City, Mt. Songshan, Haituo. |
| No. 069 | Gnaphosidae | *Kishidaia albimaculata* | China: Hebei Province, Mt. Wuling, Wuchagou, N40.56334º: E117.48740º, alt. 1112m. |
| No. 070 | Gnaphosidae | *Nomisia aussereri* | China: Xinjiang Autonomous Region, Tuoli County. |
| No. 072 | Hahniidae | *Hahnia nava* | China: Jilin Province, Hunchun City, Banjie Mountian, N42.82671º: E130.50240º, alt. 332m. |
| No. 073 | Hahniidae | *Hahnia nava* | China: Jilin Province, Hunchun City, Banjie Mountian, N42.82671º: E130.50240º, alt. 332m. |
| No. 071 | Hahniidae | *Hahnia nava* | China: Jilin Province, Hunchun City, Machuanzi Town, N42.82671º: E130.50240º, alt. 332m. |
| No. 074 | Hahniidae | *Hahnia zhejiangensis* | China: Guangxi Province, Guilin City, Huaping National Nature Reserve, N25.60600º: E109.94824º, alt. 918m. |
| No. 075 | Hahniidae | *Hahnia zhejiangensis* | China: Guangxi Province, Guilin City, Huaping National Nature Reserve, N25.60600º: E109.94824º, alt. 918m. |
| No. 076 | Hahniidae | *Hahnia zhejiangensis* | China: Guangxi Province, Guilin City, Huaping National Nature Reserve, N25.60600º: E109.94824º, alt. 918m. |
| No. 077 | Linyphiidae | *Abiskoa abiskoensis* | Finland: Pielisjärvi, Mätäsvaara, among bolders. |
| No. 078 | Linyphiidae | *Acanoides beijingensis* | China: Beijing City, Mt. Yangtai, Qiwangfen. |
| No. 080 | Linyphiidae | *Acanoides beijingensis* | China: Beijing City, Mt. Yangtai, Qiwangfen. |
| No. 079 | Linyphiidae | *Acanoides beijingensis* | China: Hebei Province, Mt. Wuling, Niangniangwa, N40.56016º: E117.49481º, alt. 1107m. |
| No. 081 | Linyphiidae | *Acanoides hengshanensis* | China: Beijing City, Mt. Yangtai, Dajue Temple. |
| No. 082 | Linyphiidae | *Acanoides hengshanensis* | China: Beijing City, Mt. Yangtai, Qiwangfen. |
| No. 083 | Linyphiidae | *Agnyphantes arboreus* | USA: Wyoming, Yellowstone Park (Grand Canyon), N40º: W110º, Wilton Ivie coll. |
| No. 084 | Linyphiidae | *Agnyphantes expunctus* | Sweden: Torne River, 1pm, Abisko National Park. |
| No. 085 | Linyphiidae | *Agyneta affinis* | Finland: Turku, Ruissalo. |
| No. 086 | Linyphiidae | *Agyneta cauta* | Finland: N40.56016º: E117.49481º |
| No. 087 | Linyphiidae | *Agyneta mollis* | Finland: Turku, Ruissalo. |
| No. 089 | Linyphiidae | *Agyneta* sp. | China: Xinjiang Autonomous Region, Kanas Lake, N48.30682º: E87.10812º, alt. 1397m. |
| No. 090 | Linyphiidae | *Agyneta* sp. | China: Heilongjiang Province, Mishan City, Xingkai Wetland Park, N45.36585º: E132.32671ºº, alt. 64m. |
| No. 091 | Linyphiidae | *Agyneta* sp. | China: Heilongjiang Province, Mishan City, Xingkai Wetland Park, N45.36585º: E132.32671ºº, alt. 64m. |
| No. 092 | Linyphiidae | *Agyneta* sp. | China: Heilongjiang Province, Mishan City, Xingkai Wetland Park, N45.36585º: E132.32671ºº, alt. 64m. |
| No. 093 | Linyphiidae | *Agyneta subtilis* | Finland: Korppoo, Lohm. |
| No. 094 | Linyphiidae | *Allomengea* sp. | China: Heilongjiang Province, Yichun City, Kangmei River, N48.266º: E129.53517º, alt. 314m. |
| No. 095 | Linyphiidae | *Anguliphantes angulipalpis* | Finland: Houtskari. |
| No. 097 | Linyphiidae | *Anguliphantes monticola* | Swizterland: Kulczyuski Saas-Tal, Wallis. |
| No. 096 | Linyphiidae | *Anguliphantes* sp. | China: Hebei Province, Chicheng County, Mt. Songshan, N40.58773º: E115.79629º, alt. 1400m. |
| No. 098 | Linyphiidae | *Anguliphantes zygius* | China: Jilin Province, Hunchun City, Dahuanggou Forestry Center, N43.06541º: E130.35750º. |
| No. 099 | Linyphiidae | *Arcuphantes arcuatulus* | USA: Oregon, Sixes (1 mile north), N42.52º: W124.30º. |
| No. 101 | Linyphiidae | *Arcuphantes dentitus* | USA: California, Plumas County, Soda Spring Gave. |
| No. 100 | Linyphiidae | *Arcuphantes fragilis* | USA: Utah, Timpanogos Park, American Fork Canyon, N40°, W111°. |
| No. 102 | Linyphiidae | *Asthenargus edentulus* | China: Xinjiang Autonomous Region, Wusulin Forest Center, N44.12847º: E84.55397º, alt. 1903m. |
| No. 103 | Linyphiidae | *Atopogyna cornupalpis* | USA: New Hampshire, Emerton Durham. |
| No. 104 | Linyphiidae | *Bathyphantes eumenis* | China: Heilongjiang Province, Yichun City, Pine Forest, N48.16189º: E129.59099º, alt. 412m. |
| No. 105 | Linyphiidae | *Bathyphantes gracilis* | China: Heilongjiang Province, Yichun City, Kangmei River, N48.266º: E129.53517º, alt. 314m. |
| No. 106 | Linyphiidae | *Bathyphantes tongluensis* | China: Hunan Province, Zhangjiajie, Wulong Village, N29.36031º: E110.41796º, alt. 950m. |
| No. 107 | Linyphiidae | *Bathyphantes tongluensis* | China: Hunan Province, Zhangjiajie, Wulong Village, N29.36031º: E110.41796º, alt. 950m. |
| No. 108 | Linyphiidae | *Bifurcia curvata* | China: Hebei Province, Xinglong County, Mt. Wuling, Longtan Fall. |
| No. 109 | Linyphiidae | *Bifurcia ramosa* | China: Sichuan Province, Tianquan County, Mt. Erlang National Forest Park. |
| No. 110 | Linyphiidae | *Bolephthyphantes* sp. | China: Xinjiang Autonomous Region, Kanas Lake, N48.71152º: E87.02639º, alt. 1394m. |
| No. 111 | Linyphiidae | *Bolyphantes alticeps* | Sweden: Medelpad, Erikglund. |
| No. 112 | Linyphiidae | *Bolyphantes* sp. | China: Xinjiang Autonomous Region, Kanas Lake, Wolong Bay, N48.62147º: E87.05192º, alt. 1342m,. |
| No. 113 | Linyphiidae | *Capsulia timushana* | China: Hunan Province, Zhangjiajie, Houhuayuan Scenic Resort, N29.3495º: E116.44265º, alt. 925m. |
| No. 114 | Linyphiidae | *Centromerus brevivulvatus* | USA: Massachusetts, Windsor, N42.31º: W73.01º. |
| No. 115 | Linyphiidae | *Centromerus denticulatus* | USA: Texas, Handeman County, Walkup Cave. |
| No. 116 | Linyphiidae | *Centromerus sylvaticus* | Finland: Turku, Kärsämäki, Pomponrahka. |
| No. 117 | Linyphiidae | *Centromerus trilobus* | China: Beijing City, Mt. Yangtai, Jinshan Temple. |
| No. 118 | Linyphiidae | *Centromerus trilobus* | China: Hebei Province, Chicheng County, Mt. Yangtai, Qiwangfen, N40.07922º: E116.06090º. |
| No. 119 | Linyphiidae | *Ceratinella* sp. | China: Heilongjiang Province, Yichun City, Tanglin Forest Center, N48.16189º: E129.59099º, alt. 412m. |
| No. 120 | Linyphiidae | *Coliinsia inerrans* | China: Heilongjiang Province, Yichun City, Tanglin Forest Center, N48.37842º: E129.53667º, alt. 407m. |
| No. 121 | Linyphiidae | *Collinsia inerrans* | China: Xinjiang Autonomous Region, Wusulin Forest Center, N44.11946º: E84.53856º, alt. 1980m. |
| No. 122 | Linyphiidae | *Cornicephalus jilinensis* | China: Jinlin Province, Changbaishan. |
| No. 123 | Linyphiidae | *Crispiphantes rhomboideus* | China: Beijing City, Mt. Yangtai, Qiwangfen. |
| No. 124 | Linyphiidae | *Crispiphantes rhomboideus* | China: Beijing City, Mt. Yangtai, Qiwangfen. |
| No. 125 | Linyphiidae | *Decipiphantes decipiens* | Finland: Utsjoki. |
| No. 126 | Linyphiidae | *Dicymbium libidinosum* | China: Heilongjiang Province, Yichun City, Pine Forest, N48.16189º: E129.59099º, alt. 412m. |
| No. 127 | Linyphiidae | *Dicymbium* sp. | China: Tibet Autonomous Region, National Route 318, 24 km, N29.78381º: EE95.69832º, alt. 3627m. |
| No. 128 | Linyphiidae | *Dicymbium tibiale* | China: Xinjiang Autonomous Region, Yili City, Narat Grassland, N43.30383º: E84.16033º, alt. 1510m. |
| No. 129 | Linyphiidae | *Doenitzius peniculus* | Japan: Tokyo, Hachioji, N35.70778º: E139.25028º. |
| No. 130 | Linyphiidae | *Doenitzius pruvus* | China: Jilin Province, Hunchun City, Banjie Mountian, N42.82671º: E130.50240º, alt. 332m. |
| No. 131 | Linyphiidae | *Doenitzius pruvus* | Japan: Tokyo, Hachioji, N35.70778º: E139.25028º. |
| No. 132 | Linyphiidae | *Drapetisca alteranda* | USA: Ontario, Island 1024, Lake Temagami, N46.59º: W80.03º. |
| No. 133 | Linyphiidae | *Drapetisca bicruis* | China: Qinghai Province, Huangyuan County. |
| No. 134 | Linyphiidae | *Drapetisca socialis* | Finland: Parainen, Mustfinnö. |
| No. 135 | Linyphiidae | *Dubiaranea penai* | Chile: Region de La Araucania (IX), Malleco Province, Tolhuaca. |
| No. 138 | Linyphiidae | *Eldonnia kayaensis* | China: Jilin Province, Hunchun City, Dahuanggou Forestry Center, N43.05153º: E130.33566º. |
| No. 136 | Linyphiidae | *Eldonnia kayaensis* | China: Jilin Province, Hunchun City, Shitou River, N42.81761º: E130.49800º, alt. 332m. |
| No. 137 | Linyphiidae | *Eldonnia kayaensis* | China: Heilongjiang Province, Dongfanghong Forest, N46.71822º: E133.52656º, alt. 152m. |
| No. 139 | Linyphiidae | *Erigone atra* | China: Xinjiang Autonomous Region, Kanas Lake, N48.71152º: E87.02639º, alt. 1394m. |
| No. 141 | Linyphiidae | *Erigone atra* | China: Xinjiang Autonomous Region, Kanas Lake, N48.71152º: E87.02639º, alt. 1394m. |
| No. 140 | Linyphiidae | *Erigone atra* | China: Jilin Province, Hunchun City, Dahuanggou Forestry Center, N43.05153º: E130.33566º. |
| No. 142 | Linyphiidae | *Erigone atra* | China: Tibet, Shannan, Konggar County, N29.27556º: E91.19869º, alt. 3496m. |
| No. 143 | Linyphiidae | *Erigone sinensis* | China: Heilongjiang Province, Yichun City, Tanglin Forest Center, N48.32447º: E129.48958º, alt. 349m. |
| No. 191 | Linyphiidae | eri-micronine sp.5 | China: Tibet, Summer meadow, N29.78486º: E95.69660º, alt. 3622m. |
| No. 144 | Linyphiidae | *Flagelliphantes bergstroemi* | Finland: Utsjoki, Kevo Puksalskaidi, stone bed (rakka). |
| No. 146 | Linyphiidae | *Floronia bucculenta* | China: Heilongjiang Province, Yichun City, Tanglin Forest Center, N48.16189º: E129.59099º, alt. 412m. |
| No. 145 | Linyphiidae | *Floronia bucculenta* | Finland: Sysmä, Virtaa & Nousianen, Pukkipalo. |
| No. 147 | Linyphiidae | *Floronia bucculenta* | China: Heilongjiang Province, Yichun City, Tanglin Forest Center, N48.16189º: E129.59099º, alt. 412m. |
| No. 148 | Linyphiidae | *Gnathonarium taczanowskii* | China: Heilongjiang Province, Yichun City, Tanglin Forest Center, N48.38052º: E129.53704º, alt. 389m. |
| No. 149 | Linyphiidae | *Gonatium japonicum* | China: Beijing City, Mt. Songshan, Haituo, N40.23915º: E115.49042º. |
| No. 150 | Linyphiidae | *Gonatium rubens* | China: Shanxi Province, Xizhou City, Mt. Wutai. |
| No. 151 | Linyphiidae | *Halorates* sp. | China: Tibet, Shannan, Konggar County, N29.27556º: E91.19869º, alt. 3496m. |
| No. 152 | Linyphiidae | *Helophora insignis* | Canada: Alberta Province, 10 miles K. W. of Whitecourt, N54.15º: W115.47º. |
| No. 153 | Linyphiidae | *Helophora orinoma* | USA: Washington, Cedar Lake, Stevens County, N48.56º: W117.36º. |
| No. 154 | Linyphiidae | *Helophora* sp. | China: Xinjiang Autonomous Region, Kanas, Hemu Village, N48.57604º: E87.44583º, alt. 172m. |
| No. 155 | Linyphiidae | *Helsdingenia ceylonica* | Ceylon: Nuwara Eliya district Hakgala, grass and litter along a mountain brook. |
| No. 156 | Linyphiidae | *Hilaira herniosa* | China: Xinjiang Autonomous Region, Kanas Lake, N48.70952º: E87.02247º, alt. 1305m. |
| No. 157 | Linyphiidae | *Hylyphantes graminicola* | China: Heilongjiang Province, Mishan City, Xingkai Wetland Park, N45.346º: E132.334º, alt. 147m. |
| No. 158 | Linyphiidae | *Improphantes complicatus* | Finland: Utsjoki, Kevo Puksalskaidi N-slope. |
| No. 159 | Linyphiidae | *Incestophantes kochiellus* | China: Xinjiang Autonomous Region, Kanas Lake, N48.70952º: E87.02247º, alt. 1305m. |
| No. 160 | Linyphiidae | *Indophantes halonatus* | China: Hubei Province, Shennongjia Forest Region, Muyuping Village, N31.7º: E110.6º. |
| No. 161 | Linyphiidae | *Indophantes kalimantanus* | Malaysia: Borneo, Sabah, Tuaran, Mount Kinabalu National Park, Panar Laban-upper forest limit, alt. 3450-3650m. |
| No. 162 | Linyphiidae | *Kaestneria pullata* | China: Heilongjiang Province, Yichun City, Pine Forest, N48.18395º: E129.58576º, alt. 399m. |
| No. 164 | Linyphiidae | *Lepthyphantes leprosus* | USA: Idaho, N.E. Fruitland, 116.44nw. |
| No. 165 | Linyphiidae | *Lepthyphantes minutus* | Finland: Nauvo. |
| No. 166 | Linyphiidae | *Lidia* sp. | China: Xinjiang Autonomous Region, Yili City, Narat, Mt. Yingxuan |
| No. 168 | Linyphiidae | *Linyphia triangularis* | China: Xinjiang Autonomous Region, Kanas, Hemu Village, N48.57177º: E87.42515º, alt. 1106m. |
| No. 169 | Linyphiidae | *Linyphia triangularis* | China: Xinjiang Autonomous Region, Tianshan Mountain Lake, N43.90878º: E88.11730º, alt. 1648m, Sep 19, 2007, L. Tu coll. |
| No. 170 | Linyphiidae | *Macrargus multesimus* | Finland: Utsjoki, Kevo Puksalskaidi N-slope. |
| No. 171 | Linyphiidae | *Macrargus rufus* | Finland: Vehkalahti. |
| No. 172 | Linyphiidae | *Macrargus rufus* | China: Xinjiang Autonomous Region, Kanas Lake, Wolong Bay, N48.62147º: E87.05192º, alt. 1342m,. |
| No. 173 | Linyphiidae | *Mansuphantes fragilis* | France: Sherman. |
| No. 174 | Linyphiidae | *Mansuphantes mansuetus* | Swizterland: Thorell Near Base 1. |
| No. 175 | Linyphiidae | *Maro minutus* | Finland: Turku, Kärsämäki, Pomponrahka. |
| No. 176 | Linyphiidae | *Maro sublestus* | Finland: Kuusamo, Juuma, |
| No. 177 | Linyphiidae | *Maso sundevallii* | China: Heilongjiang Province, Mishan City, Xingkai Wetland Park, N45.36585º: E132.32671ºº, alt. 64m. |
| No. 178 | Linyphiidae | *Megalepthyphantes nebulosus* | Höhlev, qrünau, Sacbjcn |
| No. 179 | Linyphiidae | *Megalepthyphantes occidentalis* | USA: Washington, Longmire. |
| No. 088 | Linyphiidae | *Meioneta rurestris* | Germany: Torfstgebiet, Haidgauer, Wurzacher Ried. |
| No. 180 | Linyphiidae | Mesasigone mira | China: Xinjiang Autonomous Region, Yili City, Mt. Heishan |
| No. 181 | Linyphiidae | *Mesasigone mira* | China: Beijing City, Yanqing District, Wild Duck Lake. |
| No. 182 | Linyphiidae | *Microlinyphia* sp*.* | China: Xinjiang Autonomous Region, Buerjin County, N46.68557º: E86.08693º, alt. 1014m. |
| No. 183 | Linyphiidae | *Microlinyphia* sp. | China: Xinjiang Autonomous Region, Buerjin County, N46.68557º: E86.08693º, alt. 1014m. |
| No. 184 | Linyphiidae | *Microneta aterrina* | Russia: NE Siberia 30km NW of Magadan Khasynr. |
| No. 185 | Linyphiidae | *Microneta viaria* | Finland: Turku, Kärsämäki, Pomponrahka. |
| No. 186 | Linyphiidae | *Micronetine* sp.1 | China: Guangxi Province, Guilin City, Huaping National Nature Reserve, Cujiang Station, N25.60403º: E109.90307º, alt. 857m. |
| No. 187 | Linyphiidae | *Micronetine* sp.15 | China: Xinjiang Autonomous Region, Wusulin Forest Center, N44.12847º: E84.55397º, alt. 1903m. |
| No. 188 | Linyphiidae | *Micronetine* sp.2 | China: Guangxi Province, Guilin City, Huaping National Nature Reserve, Cujiang Station, N25.60405º: E109.90138º, alt. 802m. |
| No. 189 | Linyphiidae | micronetine sp.4 | China: Inner Mongolia Autonomous Region, Alxa Left Banner, Mt. Ho-lan, South Temple, N38.67107º: E105.88975º, alt. 2200–2600m. |
| No. 190 | Linyphiidae | micronetine sp.4 | China: Inner Mongolia Autonomous Region, Alxa Left Banner, Mt. Ho-lan, South Temple, N38.67107º: E105.88975º, alt. 2200–2600m. |
| No. 192 | Linyphiidae | *Mughiphantes mughi* | Italy: Südtirol, Neves, Stausee, alt. 1880m, Larch and Pine trees, Umbrella. |
| No. 193 | Linyphiidae | *Mughiphantes nigromaculatus* | China: Beijing City, Mt. Songshan, Tangzigou Village. |
| No. 194 | Linyphiidae | *Mughiphantes nigromaculatus* | China: Hebei Province, Mt. Wuling, Baicaowa |
| No. 195 | Linyphiidae | *Mughiphantes sobrius* | Sweden: Torne River, 1pm, Abisko National Park. |
| No. 196 | Linyphiidae | *Mughiphantes* sp.B | China: Shanxi Province, Youyu County, Cangtouhe Wetland Park. |
| No. 197 | Linyphiidae | Mughiphantes sp.C | China: Xinjiang Autonomous Region, Yili City, Narat Grassland, N43.28447º: E84.22466º, alt. 1920m. |
| No. 198 | Linyphiidae | *Mughiphantes* sp.D | China: Hebei Province, Chicheng County, Mt. Songshan, N40.53711º: E115.81480º. |
| No. 200 | Linyphiidae | *Neriene emphana* | China: Hebei Province, Mt. Wuling, Wuchagou, N40.56395º: E117.48701º, alt. 1112m. |
| No. 199 | Linyphiidae | *Neriene emphana* | China: Hebei Province, Mt. Wuling, Wuchagou, N40.56395º: E117.48701º, alt. 1112m,. |
| No. 201 | Linyphiidae | *Neriene* sp.2 | China: Hebei Province, Mt. Wuling, Wuchagou, N40.56628º: E117.48611º, alt. 1176m. |
| No. 202 | Linyphiidae | *Neriene* sp.3 | China: Hebei Province, Mt. Wuling, Wuchagou, N40.56334º: E117.48740º, alt. 1112m. |
| No. 203 | Linyphiidae | *Neriene* sp.4 | China: Hebei Province, Mt. Wuling, Wuchagou, N40.56334º: E117.48740º, alt. 1112m. |
| No. 204 | Linyphiidae | *Neriene* sp.5 | China: Hebei Province, Mt. Wuling, Wuchagou, N40.56395º: E117.48701º, alt. 1112m. |
| No. 205 | Linyphiidae | *Neriene* sp.6 | China: Beijing City, Mt. Songshan, the way to Dazhuangke. |
| No. 207 | Linyphiidae | *Neriene* sp.7 | China: Guangxi Province, Guilin City, Huaping National Nature Reserve, Dingjia Bay, N25.59971º: E109.96315º, alt. 962m. |
| No. 206 | Linyphiidae | *Neriene* sp.7 | China: Guangxi Province, Guilin City, Huaping National Nature Reserve, Hongtan Station, Xiaojia Road, N25.60931º: E109.95181º, alt. 936m. |
| No. 208 | Linyphiidae | *Nesioneta ellipsoidalis* | Vietnam: Son Tay Province, Bavi District, Tan Linh Village. |
| No. 209 | Linyphiidae | *Nesioneta lepida* | Thailand: C.P., Chianmai cultural measow (yard). |
| No. 210 | Linyphiidae | *Nippononeta alpina* | China: Sichuan Province, Fenghuo Town. |
| No. 211 | Linyphiidae | *Nippononeta coreana* | China: Sichuan Province, Tianquan County, Mt. Erlang National Forest Park. |
| No. 212 | Linyphiidae | *Nippononeta kantonis* | Japan: Tokyo, Hachioji, N25.71333º: E139.24722º. |
| No. 213 | Linyphiidae | *Nippononeta kurilensis* | Russia: Sakhalin island. |
| No. 214 | Linyphiidae | *Obscuriphantes obscurus* | Finland: Somero. |
| No. 217 | Linyphiidae | *Oedothorax apicatus* | China: Xinjiang Autonomous Region, Burqin County, Irtysh River, N47.69561º: E86.85894º, alt. 469m. |
| No. 215 | Linyphiidae | *Oedothorax apicatus* | China: Xinjiang Autonomous Region, Kanas Lake, N48.09353º: E87.09648º, alt. 652m. |
| No. 216 | Linyphiidae | *Oedothorax apicatus* | China: Xinjiang Autonomous Region, Kanas Lake, N48.09353º: E87.09648º, alt. 652m. |
| No. 218 | Linyphiidae | *Oia imadatei* | China: Shanxi Province, Youyu County, Cangtouhe Wetland Park. |
| No. 219 | Linyphiidae | *Oreoneta tienshangensis* | China: Xinjiang Autonomous Region, Wusulin Forest Center, N44.12205º: E84.54845º, alt. 1965m. |
| No. 220 | Linyphiidae | *Oreonetides vaginatus* | Finland: Utsjoki. |
| No. 221 | Linyphiidae | *Oryphantes angulatus* | Finland: Turku. |
| No. 222 | Linyphiidae | *Oryphantes* sp. | China: Xinjiang Autonomous Region, Kanas, Hemu Village, N48.57177º: E87.42515º, alt. 1106m. |
| No. 223 | Linyphiidae | *Pacifiphantes zakharovi* | China: Heilongjiang Province, Yichun City, Tanglin Forest Center, N48.38043º: E129.53905º, alt. 391m. |
| No. 224 | Linyphiidae | *Palliduphantes exiguus* | Sweden: Torne River, 1pm, Abisko National Park. |
| No. 225 | Linyphiidae | *Palliduphantes pallidus* | Russia. |
| No. 226 | Linyphiidae | *Parameioneta bilobata* | China: Fujian Province, Jiangle County, Mt. Longqi. |
| No. 227 | Linyphiidae | *Parameioneta spicata* | Thailand. |
| No. 228 | Linyphiidae | *Parawubanoides unicornis* | China: Inner Mongolia Autonomous Region, Lov Aimalu, N48.36667º: E106.30000º, alt. 1100m. |
| No. 229 | Linyphiidae | *Poeciloneta bihamatus* | Labeled as “nuntfast, 2ne. Jun 10, 1914 V.H.E” |
| No. 230 | Linyphiidae | *Poeciloneta variegata* | Germany: Bavaria, Swabia, douglas fir (Pseudotsuga menziesii) forest near Krumbach, N48.0994º: E10.3464º, alt. 550m. |
| No. 231 | Linyphiidae | *Rhabdoria diluta* | France: Fountainbleau. |
| No. 232 | Linyphiidae | *Ryojius nanyuensis* | China: Henan Province, Baotianman Nature Reserve. |
| No. 233 | Linyphiidae | *Saaristoa abnormis* | Germany: Bavaria, Middle Franconia, forest border 'Kronberg' near Feuchtwangen, N49.19º: E10.299º, alt. 470m. |
| No. 234 | Linyphiidae | *Saaristoa higoensis* | Japan: Kumamoto Prefecture, Itsuki-mura. |
| No. 235 | Linyphiidae | *Saaristoa* sp. | China: Guangxi Province, Guilin City, Huaping National Nature Reserve, Hongtan Station, N25.60688º: E109.94809º, alt. 874m. |
| No. 236 | Linyphiidae | *Saaristoa* sp. | China: Guangxi Province, Guilin City, Huaping National Nature Reserve, Cujiang Station, N25.59757º: E109.90701º, alt. 798m. |
| No. 237 | Linyphiidae | *Saaristoa* sp. | China: Guangxi Province, Guilin City, Huaping National Nature Reserve, Cujiang Station, N25.59757º: E109.90701º, alt. 798m. |
| No. 238 | Linyphiidae | *Savignia* sp.1 | China: Hebei Province, Chicheng County, Mt. Songshan, Haituo, N40.56251º: E115.81273º. |
| No. 240 | Linyphiidae | *Savignia* sp.2 | China: Xinjiang Autonomous Region, Yili City, Narat Grassland, N43.28447º: E84.22466º, alt. 1920m. |
| No. 239 | Linyphiidae | *Savignia* sp.2 | China: Xinjiang Autonomous Region, Yili City, Narat Grassland, N43.28447º: E84.22466º, alt. 1920m. |
| No. 241 | Linyphiidae | *Solenysa lanyuensis* | China: Taiwan Island, Taidong City, Lanyu, N22.70°: E121.10°. |
| No. 244 | Linyphiidae | *Solenysa longqiensis* | China: Fujian Province, Jiangle County, Mt. Longqi, Yujiaping Town, N26.70º: E 117.40º. |
| No. 243 | Linyphiidae | *Solenysa longqiensis* | Japan: Honshu, Aichi Prefecture, Okazaki-shi, Okuyamada-cho, Mt. Murazumi-yama, alt. 200–250 m. |
| No. 245 | Linyphiidae | *Solenysa macrodonta* | Japan: Ko-ajiro, Miura, Kanagawa, N35.16742º: E139.62750º. |
| No. 246 | Linyphiidae | *Solenysa mellotteei* | Japan: Nishi-ueda, Takamatsu, Kagawa, N34.22028º: E134.07695º, alt. 130m, |
| No. 247 | Linyphiidae | *Solenysa partibilis* | Japan: Ohshimizu, Maibara, Shiga. |
| No. 248 | Linyphiidae | *Solenysa protrudens* | China: Zhejiang Province, Hangzhou Zhongshan Park. |
| No. 249 | Linyphiidae | *Solenysa reflexilis* | Japan: Kumamoto City, N32.37288º: E130.85889º, alt. 590m. |
| No. 250 | Linyphiidae | *Solenysa retractilis* | China: Sichuan Province, Longdong Town, Baoxing County, N30.30º: E102.80º. |
| No. 251 | Linyphiidae | *Solenysa* sp.n. | China: Chongqing, Yiyang Tujia and Miao Autonomous County, Taohuayuan Scenic Spot, N28.85097°, E108.75037°, alt. 995m. |
| No. 252 | Linyphiidae | *Solenysa tianmushana* | China: Zhejiang Province, Hangzhou City, Mt. East Tianmu, N30.33615º: E119.50257º, alt. 349m. |
| No. 253 | Linyphiidae | *Solenysa trunciformis* | Japan: Yunotsu Town, Shimane, N35.08944º: E132.41167º, alt. 240m. |
| No. 254 | Linyphiidae | *Solenysa wulingensis* | China: Hunan Province, Zhangjiajie, Houhuayuan Scenic Resort, N29. 34950º: E110.44365º, alt. 925m. |
| No. 242 | Linyphiidae | *Solenysa yangmingshana* | China: Taiwan Island, Taibei City, Mt. Yangmingshan, N26.70°: E117.40°. |
| No. 256 | Linyphiidae | *Stemonyphantes* *abatensis* | China: Xinjiang Autonomous Region, Wusulin Forest Center, N44.12847º: E84.55397º, alt. 1903m. |
| No. 255 | Linyphiidae | *Stemonyphantes abatensis* | China: Xinjiang Autonomous Region, Wusulin Forest Center, N44.12205º: E84.54845º, alt. 1965m. |
| No. 257 | Linyphiidae | *Styloctetor stativa* | China: Xinjiang Autonomous Region, Kanas, Hemu Village, N48.57177º: E87.42515º, alt. 1106m. |
| No. 258 | Linyphiidae | *Syedra gracilis* | Germany: MZT AM 1064 |
| No. 259 | Linyphiidae | *Syedra oii* | China: Zhejiang Province, Hangzhou City, Mt. Tianmu. |
| No. 260 | Linyphiidae | *Tallusia experta* | Finland: Naantali. |
| No. 261 | Linyphiidae | *Tapinopa bilineata* | Ramsey, N.J. |
| No. 262 | Linyphiidae | *Tapinopa longidens* | Finland: Turku. |
| No. 264 | Linyphiidae | *Tapinopa* sp. | China: Hebei Province, Mt. Wuling, Niangniangwa, N40.56016º: E117.49481º, alt. 1107m. |
| No. 263 | Linyphiidae | *Tapinopa* sp. | China: Beijing City, Mt. Songshan, Tangzigou Village. |
| No. 265 | Linyphiidae | *Tenuiphantes mengei* | Azerbaijan: Apsheron, Peninsula, Baku, Ganly-Gyol L, N40.35767°: E49.82267°. |
| No. 266 | Linyphiidae | Tenuiphantes mengei | China: Xinjiang Autonomous Region, Kanas Lake, Wolong Bay, N48.62147º: E87.05192º, alt. 1342m. |
| No. 267 | Linyphiidae | *Tenuiphantes* sp. | China: Xinjiang Autonomous Region, Kanas Lake, Wolong Bay, N48.62147º: E87.05192º, alt. 1342m. |
| No. 268 | Linyphiidae | *Tenuiphantes tenebricola* | Finland: Vehkalahti. |
| No. 269 | Linyphiidae | *Tenuiphantes tenuis* | Chile: Oaorno. |
| No. 270 | Linyphiidae | *Tenuiphantes tenuis* | Azerbaijan: Nakhchevan area, env. of Bichenek vill, N39. 52827°: E45.77667°, alt. 2000m. |
| No. 271 | Linyphiidae | *Ternatus malleatus* | China: Guangxi Province, Guilin City, Huaping National Nature Reserve, Hongtan Station, Xiaojia Road, N25.60931º: E109.95181º, alt. 936m. |
| No. 272 | Linyphiidae | *Ternatus siculus* | China: Hunan Province, Zhangjiajie, N29.34719º: E110.43433º, alt. 938m. |
| No. 273 | Linyphiidae | *Ummeliata insecticeps* | China: Beijing City, Yanqing District, Wild Duck Lake. |
| No. 274 | Linyphiidae | *Vagiphantes vaginatus* | China: Xinjiang Autonomous Region, Wusulin Forest Center, N44.12847º: E84.55397º, alt. 1903m. |
| No. 275 | Linyphiidae | *Wubanoides* sp. | China: Xinjiang Autonomous Region, Kanas, N48.70926º: E87.02947º, alt. 1348m. |
| No. 163 | Linyphiidae | *Lepthyphantes luteipes* | China: Xinjiang Autonomous Region, Kanas Lake, N48.70952º: E87.02247º, alt. 1305m. |
| No. 276 | Lycosidae | *Alopecosa licenti* | China: Beijing City, Mt. Songshan, Haituo. |
| No. 277 | Lycosidae | *Lycosa wulsini* | China: Beijing City, Mt. Songshan, Haituo. |
| No. 278 | Lycosidae | *Lycosa wulsini* | China: Beijing City, Mt. Songshan, Haituo. |
| No. 279 | Lycosidae | *Lycosa wulsini* | China: Beijing City, Mt. Songshan, Haituo. |
| No. 280 | Lycosidae | *Lycosa wulsini* | China: Beijing City, Mt. Songshan, Haituo. |
| No. 281 | Lycosidae | *Pardosa chionophila* | China: Beijing City, Mt. Songshan, Haituo. |
| No. 292 | Lycosidae | *Pardosa* sp.2 | China: Shanxi Province, Wuzhou City, Wutai Mountain. |
| No. 293 | Lycosidae | *Pardosa* sp.2 | China: Shanxi Province, Wuzhou City, Wutai Mountain. |
| No. 298 | Lycosidae | *Pirata* sp. | China: Guizhou Province, Zunyi City, Red Army Mountain, N27.68907º: E106.90571º. |
| No. 299 | Lycosidae | *Trochosa ruricola* | China: Beijing City, Mt. Songshan, Haituo. |
| No. 300 | Miturgidae | *Zora lyriformis* | China: Beijing City, Mt. Songshan, Haituo. |
| No. 301 | Miturgidae | *Zora* sp.1 | China: Xinjiang Autonomous Region, Kanas, Tochigi Village, N48.57604º: E87.44583º, alt. 1720m. |
| No. 302 | Miturgidae | *Zora* sp.2 | China: Tibet, The road from Shannan to Lhasa, N29.24733º: E91.70889º, alt. 3566m. |
| No. 048 | Nephilidae | *Trichonephila clavata* | China: Beijing City, Mt. Yangtai, Jinshan Temple, N40.0675º: E116.0811º, alt. 372m. |
| No. 047 | Nephilidae | *Trichonephila clavata* | China: Beijing City, Mt. Yangtai, Jinshan Temple, N40.0675º: E116.0811º, alt. 372m. |
| No. 049 | Nephilidae | *Trichonephila clavata* | China: Hubei Province, Shennongjia Forestry District. |
| No. 310 | Nesticidae | *Nesticella mogera* | China: Beijing City, Mt. Songshan, Haituo. |
| No. 312 | Oecobiidae | *Oecobius* sp. | China: Beijing City, Capital Normal University campus. |
| No. 313 | Philodromidae | *Philodromus* sp. | China: Beijing City, Mt. Songshan, Haituo. |
| No. 314 | Philodromidae | *Thanatus* sp. | China: Tibet, Linzhi, The intersection of the Nyang River and YarluZangbu rivers, N29.43353º: E94.45307º, alt. 2940m. |
| No. 315 | Philodromidae | *Thanatus* sp. | China: Tibet, Linzhi, The intersection of the Nyang River and YarluZangbu rivers, N29.43353º: E94.45307º, alt. 2940m. |
| No. 316 | Philodromidae | *Tibellus tenellus* | China: Hebei Province, Mt. Wuling, Wuchagou, N40.56395º: E117.48701º, alt. 1112m. |
| No. 318 | Phrurolithidae | *Orthobula crucifera* | China: Beijing City, Mt. Yangtai, Jinshan Temple, N40.0675º: E116.0811º, alt. 372m. |
| No. 319 | Phrurolithidae | *Otacilia komurai* | China: Guangxi Province, Guilin City, Huaping National Nature Reserve, N25.60600º: E109.94824º, alt. 918m. |
| No. 320 | Phrurolithidae | *Otacilia komurai* | China: Guangxi Province, Guilin City, Huaping National Nature Reserve, N25.60822º: E109.95104º, alt. 903m. |
| No. 321 | Pimoidae | *Putaoa huaping* | China: Guangxi Province, Guilin City, Huaping National Nature Reserve, Hongtan Station, N25.60876º: E109.94865º, alt. 932m. |
| No. 322 | Pimoidae | *Putaoa huaping* | China: Guangxi Province, Guilin City, Huaping National Nature Reserve, Cujiang Station, N25.60405º: E109.90138º, alt. 802m. |
| No. 324 | Salticidae | *Phintella popovi* | China: Jilin Province, Hunchun City, Banjie Mountian, N42.82671º: E130.50240º, alt. 332m. |
| No. 325 | Salticidae | *Portia quei* | China: Guizhou Province, Anshun County, Tianxing Bridge, N25.95023º: E105.67627º, alt. 829m. |
| No. 326 | Salticidae | *Pseudeuophrys* sp. | China: Tibet, National Road 318 4249K， Coniferous mixed forest, N29.76587º: E94.24681º, alt. 3018m. |
| No. 328 | Salticidae | undet. sp. | China: Beijing City, Mt. Songshan, Haituo. |
| No. 327 | Salticidae | undet. sp. | China: Beijing City, Capital Normal University campus. |
| No. 329 | Salticidae | *Yaginumaella* sp. | China: Tibet, Highway S306 87K, N29.22865º: E94.24093º, alt. 2965m. |
| No. 330 | Salticidae | *Yaginumaella* sp. | China: Tibet, Highway S306 87K, N29.22865º: E94.24093º, alt. 2965m. |
| No. 331 | Salticidae | *Yllenus erzinensis* | China: Inner Mongolia Autonomous Region, the way to Keerlun Sumu, N48.45434º: E116.4324º, alt. 576m. |
| No. 332 | Sparassidae | *Pseudopoda* sp. | China: Tibet, Highway S306 87K, N29.22865º: E94.24093º, alt. 2965m. |
| No. 379 | Tetragnathidae | *Diphya* sp. | China: Guangxi Province, Guilin City, Huaping National Nature Reserve, N25.60405º: E109.90138º, alt. 802m. |
| No. 354 | Tetragnathidae | *Diphya wulingensis* | China: Hebei Province, Mt. Wuling, Wuchagou, N40.56628º: E117.48611º, alt. 1176m. |
| No. 355 | Tetragnathidae | *Diphya wulingensis* | China: Hebei Province, Mt. Wuling, Wuchagou, N40.56628º: E117.48611º, alt. 1176m. |
| No. 356 | Tetragnathidae | *Diphya wulingensis* | China: Hebei Province, Mt. Wuling, Wuchagou, N40.56628º: E117.48611º, alt. 1176m. |
| No. 347 | Tetragnathidae | *Diphya wulingensis* | China: Hebei Province, Mt. Wuling, Wuchagou, N40.56628º: E117.48611º, alt. 1176m. |
| No. 348 | Tetragnathidae | *Diphya wulingensis* | China: Hebei Province, Mt. Wuling, Wuchagou, N40.56628º: E117.48611º, alt. 1176m. |
| No. 349 | Tetragnathidae | *Diphya wulingensis* | China: Hebei Province, Mt. Wuling, Wuchagou, N40.56628º: E117.48611º, alt. 1176m. |
| No. 350 | Tetragnathidae | *Diphya wulingensis* | China: Hebei Province, Mt. Wuling, Wuchagou, N40.56628º: E117.48611º, alt. 1176m. |
| No. 351 | Tetragnathidae | *Diphya wulingensis* | China: Hebei Province, Mt. Wuling, Wuchagou, N40.56628º: E117.48611º, alt. 1176m. |
| No. 352 | Tetragnathidae | *Diphya wulingensis* | China: Hebei Province, Mt. Wuling, Wuchagou, N40.56628º: E117.48611º, alt. 1176m. |
| No. 353 | Tetragnathidae | *Diphya wulingensis* | China: Hebei Province, Mt. Wuling, Wuchagou, N40.56628º: E117.48611º, alt. 1176m. |
| No. 345 | Tetragnathidae | *Diphya wulingensis* | China: Hebei Province, Mt. Wuling, Wuchagou, N40.56334º: E117.48740º, alt. 1112m. |
| No. 342 | Tetragnathidae | *Diphya wulingensis* | China: Hebei Province, Mt. Wuling, Wuchagou, N40.56395º: E117.48701º, alt. 1112m. |
| No. 340 | Tetragnathidae | *Diphya wulingensis* | China: Hebei Province, Mt. Wuling, Wuchagou, N40.56628º: E117.48611º, alt. 1176m. |
| No. 341 | Tetragnathidae | *Diphya wulingensis* | China: Hebei Province, Mt. Wuling, Wuchagou, N40.56395º: E117.48701º, alt. 1112m. |
| No. 346 | Tetragnathidae | *Diphya wulingensis* | China: Heilongjiang Province, Dongfanghong Forest Farm Qingshan Forest Farm, N46.47835º: E133.31142º, alt. 125m. |
| No. 344 | Tetragnathidae | *Diphya wulingensis* | China: Hebei Province, Mt. Wuling, Wuchagou, N40.56628º: E117.48611º, alt. 1176m. |
| No. 343 | Tetragnathidae | *Diphya wulingensis* | China: Hebei Province, Mt. Wuling, Niangniangwa, N40.56016º: E117.49481º, alt. 1107m. |
| No. 361 | Tetragnathidae | *Leucauge* sp. | China: Guizhou Province, Anshun County, Tianshun Bridge, N25.95023º: E105.67271º, alt. 829m. |
| No. 359 | Tetragnathidae | *Leucauge* sp. | China: Guizhou Province, Anshun County, N25.99743º: E105.67271º, alt. 1061m. |
| No. 360 | Tetragnathidae | *Leucauge* sp. | China: Guizhou Province, Anshun County, N25.99743º: E105.67271º, alt. 1061m. |
| No. 370 | Tetragnathidae | *Metleucauge yunohamensis* | China: Hebei Province, Mt. Wuling, Niangniangwa, N40.56016º: E117.49481º, alt. 1107m. |
| No. 371 | Tetragnathidae | *Metleucauge yunohamensis* | China: Hebei Province, Mt. Wuling, Niangniangwa, N40.56016º: E117.49481º, alt. 1107m. |
| No. 368 | Tetragnathidae | *Metleucauge yunohamensis* | China: Hebei Province, Mt. Wuling, Niangniangwa, N40.56016º: E117.49481º, alt. 1107m. |
| No. 369 | Tetragnathidae | *Metleucauge yunohamensis* | China: Hebei Province, Mt. Wuling, Niangniangwa, N40.56016º: E117.49481º, alt. 1107m. |
| No. 380 | Theridiidae | *Enoplognatha ovata* | Poland: The Bug River Valley, N52.2684º: E23.1673º, alt. 122m. |
| No. 382 | Theridiidae | *Paidiscura subpallens* | China: Guizhou Province, Xifeng Concentration Camp, N27.03669º: E106.73502º, alt. 890m. |
| No. 381 | Theridiidae | *Paidiscura subpallens* | China: Guizhou Province, Xifeng Concentration Camp, N27.03669º: E106.73502º, alt. 890m. |
| No. 384 | Theridiidae | *Parasteatoda tepidariorum* | China: Guizhou Province, Tianlong Tunpu, Mt. Tiantai, N26.34566º: E106.17463º, alt. 1398m. |
| No. 385 | Theridiidae | *Parasteatoda tepidariorum* | China: Guizhou Province, Anshun County, Huangguoshu, N25.98668º: E105.65593º. |
| No. 386 | Theridiidae | *Parasteatoda tepidariorum* | China: Guizhou Province, Anshun County, Huangguoshu, N25.98668º: E105.65594º. |
| No. 387 | Theridiidae | *Parasteatoda tepidariorum* | China: Guizhou Province, Anshun County, Huangguoshu, N25.98668º: E105.65595º. |
| No. 394 | Theridiidae | *Phycosoma sinica* | China: Beijing City, Mt. Yangtai, Jinshan Temple, N40.0675º: E116.0811º, alt. 372m. |
| No. 388 | Theridiidae | *Phylloneta impressa* | China: Ningxia Hui Autonomous Region, Liupan Mountain. |
| No. 389 | Theridiidae | *Robertus* sp. | China: Xinjiang Autonomous Region, Yili City, Sayram Lake, N44.49413º: E81.18502º, alt. 2073m. |
| No. 391 | Theridiidae | *Steatoda mainlingensis* | China: Tibet, Highway S306 87K, N29.19657º: E94.09316º, alt. 2966m. |
| No. 392 | Theridiidae | *Thymoites bellissimus* | China: Heilongjiang Province, Yichun, Xinqing District, Tanglin Forest Farm, N48.38043º: E129.53905º, alt. 391m. |
| No. 393 | Theridiidae | undet. sp. | China: Beijing City, Mt. Songshan, Tangzigou Village. |
| No. 395 | Theridiidae | *Yaginumena castrata* | China: Hebei Province, Mt. Wuling, Niangniangwa, N40.56016º: E117.49481º, alt. 1107m. |
| No. 396 | Theridiidae | *Yaginumena castrata* | China: Hebei Province, Mt. Wuling, Niangniangwa, N40.56016º: E117.49481º, alt. 1107m. |
| No. 401 | Thomisidae | *Misumena vatia* | China: Hebei Province, Mt. Wuling, Wuchagou, N40.56395º: E117.48701º, alt. 1112m. |
| No. 403 | Thomisidae | *Ozyptila praticola* | China: Beijing City, Mt. Songshan, Haituo. |
| No. 402 | Thomisidae | *Ozyptila praticola* | China: Xinjiang Autonomous Region, Yili City, Nalati, N43.30383º: E84.16033º, alt. 1510m. |
| No. 400 | Thomisidae | *Tmarus rimosus* | China: Hebei Province, Mt. Wuling, Wuchagou, N40.56334º: E117.48740º, alt. 1112m. |
| No. 404 | Thomisidae | *Xysticus emenfoni* | China: Beijing City, Mt. Songshan, Haituo. |
| No. 405 | Thomisidae | *Xysticus emenfoni* | China: Beijing City, Mt. Songshan, Haituo. |
| No. 406 | Thomisidae | *Xysticus emertoni* | China: Beijing City, Mt. Songshan, Haituo. |
| No. 407 | Thomisidae | *Xysticus* sp.1 | China: Beijing City, Mt. Songshan, Tangzigou Village. |
| No. 408 | Thomisidae | *Xysticus* sp.2 | China: Beijing City, Mt. Songshan, Tangzigou Village. |
| No. 409 | Titanoecidae | *Titanoeca* sp. | China: Tibet, 318 Sichuan-Tibet Highway 4606K, N29.68237º: E91.41683º, alt. 3773m. |
| No. 410 | Uloboridae | *Octonoba sinensis* | China: Hebei Province, Hebei University. |
