# Supplementary material for: A spider mating plug functions to protect sperm
Source: PLoS One. 2024 Mar 29;19(3):e0301290. doi: 10.1371/journal.pone.0301290 (PMC10980215; doi:10.1371/journal.pone.0301290)
Supplement: Attachment — Submitted filename: PONE-D-22-34374_reviewer_FAP.pdf [file pone.0301290.s004.pdf]

PLOS ONE

A spider mating plug functions to protect sperm

--Manuscript Draft--

**Manuscript Number:**	PONE-D-22-34374
**Article Type:**	Research Article
**Full Title:**	A spider mating plug functions to protect sperm
**Short Title:**	Mating plug functions to protect sperm
**Corresponding Author:**	Lihong Tu, PhD<br>Capital Normal University<br>Beijing, CHINA
**Keywords:**	mating plug; secretion plug; genital plug; sperm plug; entelegyne spider; entelegyne spermatheca; spermathecal morphology; epigynal tract; mating strategy; sperm protection mechanism
**Abstract:**	Mating plugs in animals are ubiquitous and are commonly interpreted to be products of mating strategies. In spiders, however, mating plugs may also take on functions beyond female remating prevention. Due to the vagaries of female genital (spermathecal) anatomy, most spiders face the problem of having to secure additional, non-anatomical, protection for transferred sperm. Here, we test the hypothesis that mating plugs, rather than (or in addition to) being adaptations for mating strategies, may serve as sperm protection mechanism. Our comparative study on female genital morphologies in entelegyne spiders confirms the necessity of a sperm protection mechanism. We also study detailed morphology of epigynal tracts in the spider Diphya wulingensis using 3D-reconstruction based on semi thin histological series section. In this species, we hypothesize that two distinct types of mating plug, the sperm plug and the secretion plug, serve different functions. Morphological details support this: sperm plugs are formed on a modified spermathecal wall by the spilled sperm, and function as a temporary protection mechanism to prevent sperm from leaking and desiccating, while secretion plugs function in postcopulation both as a permanent protection mechanism, and to prevent additional mating. Furthermore, with the modified spermathecal wall of S2 stalk, the problem of shunt for sperm input and output, and the possibility of female multiple mating have been resolved. Variation in spermathecal morphology also suggests that the problem of sperm protection might be resolved in different ways in spiders. Considering mating plugs of varying shapes and origins in the vast morphospace of spiders, we conclude that mating plugs might serve different purposes that relate both to mating strategies, as well as to sperm protection.
**Order of Authors:**	He Jiang
	Yongjia Zhan
	Qingqing Wu
	Huitao Zhang
	Matjaž Kuntner
	Lihong Tu, PhD
**Additional Information:**	
**Question**	**Response**
**Financial Disclosure**<br><br>Enter a financial disclosure statement that describes the sources of funding for the work included in this submission. Review the submission guidelines for detailed requirements. View published research articles from *PLOS ONE* for specific	Funding for this research is provided by the National Natural Sciences Foundation of China NSFC-31572244 and NSFC-31872188 to LT, NSFC-61671311 to HZ. The funders had no role in study design, data collection and analysis, decision to publish, or preparation of the manuscript.

Powered by Editorial Manager® and ProduXion Manager® from Aries Systems Corporation

examples.<br><br>This statement is required for submission and **will appear in the published article** if the submission is accepted. Please make sure it is accurate.<br><br>**Unfunded studies**<br>Enter: *The author(s) received no specific funding for this work.*<br><br>**Funded studies**<br>Enter a statement with the following details:<br>• Initials of the authors who received each award<br>• Grant numbers awarded to each author<br>• The full name of each funder<br>• URL of each funder website<br>• Did the sponsors or funders play any role in the study design, data collection and analysis, decision to publish, or preparation of the manuscript?<br>• **NO** - Include this sentence at the end of your statement: *The funders had no role in study design, data collection and analysis, decision to publish, or preparation of the manuscript.*<br>• **YES** - Specify the role(s) played.<br><br>* typeset	
**Competing Interests**<br><br>Use the instructions below to enter a competing interest statement for this submission. On behalf of all authors, disclose any competing interests that could be perceived to bias this work—acknowledging all financial support and any other relevant financial or non-financial competing interests.<br><br>This statement is required for submission and **will appear in the published article** if the submission is accepted. Please make sure it is accurate and that any funding sources listed in your Funding Information later in the submission form are also declared in your Financial Disclosure statement.	the authors have declared that no competing interests exist.

View published research articles from *PLOS ONE* for specific examples.<br><br>**NO authors have competing interests**<br><br>Enter: *The authors have declared that no competing interests exist.*<br><br>**Authors with competing interests**<br><br>Enter competing interest details beginning with this statement:<br><br>*I have read the journal's policy and the authors of this manuscript have the following competing interests: [insert competing interests here]*<br><br>* typeset	
**Ethics Statement**<br><br>Enter an ethics statement for this submission. This statement is required if the study involved:<br><br>• Human participants<br>• Human specimens or tissue<br>• Vertebrate animals or cephalopods<br>• Vertebrate embryos or tissues<br>• Field research<br><br>Write "N/A" if the submission does not require an ethics statement.<br><br>General guidance is provided below. Consult the submission guidelines for detailed instructions. **Make sure that all information entered here is included in the Methods section of the manuscript.**	N/A

**Format for specific study types**<br><br>**Human Subject Research (involving human participants and/or tissue)**<br>• Give the name of the institutional review board or ethics committee that approved the study<br>• Include the approval number and/or a statement indicating approval of this research<br>• Indicate the form of consent obtained (written/oral) or the reason that consent was not obtained (e.g. the data were analyzed anonymously)<br><br>**Animal Research (involving vertebrate animals, embryos or tissues)**<br>• Provide the name of the Institutional Animal Care and Use Committee (IACUC) or other relevant ethics board that reviewed the study protocol, and indicate whether they approved this research or granted a formal waiver of ethical approval<br>• Include an approval number if one was obtained<br>• If the study involved *non-human primates*, add *additional details* about animal welfare and steps taken to ameliorate suffering<br>• If anesthesia, euthanasia, or any kind of animal sacrifice is part of the study, include briefly which substances and/or methods were applied<br><br>**Field Research**<br><br>Include the following details if this study involves the collection of plant, animal, or other materials from a natural setting:<br>• Field permit number<br>• Name of the institution or relevant body that granted permission	
**Data Availability**<br><br>Authors are required to make all data underlying the findings described fully available, without restriction, and from the time of publication. PLOS allows rare exceptions to address legal and ethical concerns. See the PLOS Data Policy and FAQ for detailed information.	Yes - all data are fully available without restriction

A Data Availability Statement describing where the data can be found is required at submission. Your answers to this question constitute the Data Availability Statement and **will be published in the article**, if accepted.<br><br>**Important:** Stating 'data available on request from the author' is not sufficient. If your data are only available upon request, select 'No' for the first question and explain your exceptional situation in the text box.<br><br>Do the authors confirm that all data underlying the findings described in their manuscript are fully available without restriction?	
**Describe where the data may be found in full sentences. If you are copying our sample text, replace any instances of XXX with the appropriate details.**<br><br>• If the data are **held or will be held in a public repository**, include URLs, accession numbers or DOIs. If this information will only be available after acceptance, indicate this by ticking the box below. For example: *All XXX files are available from the XXX database (accession number(s) XXX, XXX.*).<br>• If the data are all contained **within the manuscript and/or Supporting Information files**, enter the following: *All relevant data are within the manuscript and its Supporting Information files.*<br>• If neither of these applies but you are able to provide **details of access elsewhere**, with or without limitations, please do so. For example:<br><br>*Data cannot be shared publicly because of [XXX]. Data are available from the XXX Institutional Data Access / Ethics Committee (contact via XXX) for researchers who meet the criteria for access to confidential data.*<br><br>*The data underlying the results presented in the study are available from (include the name of the third party*	All relevant data are within the manuscript and its Supporting Information files.

and contact information or URL).<br>• This text is appropriate if the data are owned by a third party and authors do not have permission to share the data.<br><br>* typeset	
Additional data availability information:	

Manuscript

Click here to access/download;Manuscript;MS-submitted version.docx

A spider mating plug functions to protect sperm

He Jiang[1], Yongjia Zhan[1], Qingqing Wu[2], Huitao Zhang[3], Matjaž Kuntner[4,5], Lihong Tu[1*]

[1] College of Life Sciences, Capital Normal University, Beijing, P. R. China

[2] Lang Yue Campus of Beijing 12th High School, Beijing, P. R. China

[3] Beijing Advanced Innovation Center for Imaging Technology, Capital Normal University, Beijing, P. R. China

[4] Department of Organisms and Ecosystems Research, National Institute of Biology, Ljubljana, Slovenia

[5] Jovan Hadži Institute of Biology, ZRC SAZU, Ljubljana, Slovenia

* Corresponding author

E-mail: tulh@cnu.edu.cn (LT)

Abstract

Mating plugs in animals are ubiquitous and are commonly interpreted to be products of mating strategies. In spiders, however, mating plugs may also take on functions beyond female remating prevention. Due to the vagaries of female genital (spermathecal) anatomy, most spiders face the problem of having to secure additional, non-anatomical, protection for transferred sperm. Here, we test the hypothesis that mating plugs, rather than (or in addition to) being adaptations for mating strategies, may serve as sperm protection mechanism. Our comparative study on female genital morphologies in entelegyne spiders confirms the necessity of a sperm protection mechanism. We also study detailed morphology of epigynal tracts in the spider *Diphya wulingensis* using 3D-reconstruction based on semi thin histological series section. In this species, we hypothesize that two distinct types of mating plug, the sperm plug and the secretion plug, serve different functions. Morphological details support this: sperm plugs are formed on a modified spermathecal wall by the spilled sperm, and function as a temporary protection mechanism to prevent sperm from leaking and desiccating, while secretion plugs function in postcopulation both as a permanent protection mechanism, and to prevent additional mating. Furthermore, with the modified spermathecal wall of S_2 stalk, the problem of shunt for sperm input and output, and the possibility of female multiple mating have been resolved. Variation in spermathecal morphology also suggests that the problem of sperm protection might be resolved in different ways in spiders. Considering mating plugs of varying shapes and origins in the vast morphospace of spiders, we conclude that mating plugs might serve different purposes that relate both to mating strategies, as well as to sperm protection.

INTRODUCTION

Mating plugs in animals are ubiquitous and are commonly interpreted to be products of mating strategies [1−5], while in spiders they may function beyond female remating prevention. Internal fertilization makes the copulation process in spiders separated from fertilization. Female genitals are equipped with spermathecae, paired organs that store sperm for prolonged times between copulation and fertilization [6−8]. These organs must be under selective pressures that relate to polygamy, as well as to sperm protection. Such selection is manifested in a diversity of shapes and forms in mating strategies and in sperm protection mechanism. Mating plugs are common in spiders, and are usually explained to function as a product of male, or female, mating strategies [7,9 −12] since they provide physical blockage that prevents additional mating. However, mating plugs could plausibly also provide sperm protection [13]. These two functional hypotheses, of course, need not be mutually exclusive.

By far most species of spiders are described as entelegyne, meaning that their copulatory and fertilization ducts to and from the spermathecae are unlinked [14]. In stark contrast to the alternative condition (haplogyn[illegible] the entelegyne gestalt poses a unique challenge related to sperm protection. Namely, the entelegyne spermatheca has separate openings for sperm inflow and outflow, respectively, and strongly sclerotized walls of these openings render the spermathecal entrances perpetually open [7,14,15]. Spermathecae store sperm for extended periods of time only to be released during oviposition [6–8]. These facts pose certain risks for

the viability of sperm lodged in spermathecae: sperm leakage and desiccation during post copulation, and sperm backflow when being released. Structural features to avoid these risks, such as potential valves that would close the spermathecal entrances, would be logically expected, but remain unknown in spiders. Mating plugs formed during copulation in many spiders may present a potential resolution to avoid the risks of sperm storage [13].

Previous studies of mating plugs in spiders have not reached a general conclusion as to their function. Mating plugs refer to either genital pieces (hereafter genital plug) or hardened secretions (hereafter secretion plug) lodged in copulatory openings during or after copulation. Generally, it is thought that mating plugs function as a mating strategy to protect male's paternity (see review by [7], or as means for female rejection of unwanted, excessive copulations [12]. Despite some support for the mating strategy hypothesis through behavioral trials in several spiders [9,10,16,17], counter-examples nevertheless abound [7,18], especially in some cases the mating plugs are formed under collaborations of both sexes [19,20] . Furthermore, the secretion plugs in some spiders not only block copulatory openings, but also the groove slits on the epigynal surface, even the whole epigynal plate (e.g., *Neriene emphana* (Linyphiidae), Tian et al. unpublished data; *Leucauge argyra* (Tetragnathidae), [21]: fig 148A; *Metleucauge eldorado* (Tetragnathidae), [21]: fig 79A; *Tengella radiata* (Tengellidae), [22]: fig 171D; *Toxopsiella minuta* (Cycloctenidae), [22]: fig 172A). These imply that mating plugs may also take other functions beyond being an obstacle for mating. Considering the lack of general evidence for the paternity protection function [23,24], Huber [13] proposed an alternative hypothesis, that plugs might to prevent the sperm lodged in the spermatheca from

leakage and desiccation. So far, the only empirical evidence for Huber's sperm protection hypothesis is attributed to the case of *Neriene emphana*, a linyphiid spider (Tian et al. unpublished data). However, the genital morphology and mating behavior of *Neriene* species are quite unique [25], atypical of entelegyne spiders. Thus, the sperm protection hypothesis has yet to be tested.

Of course, the mating strategy and the sperm protection hypotheses need not be mutually exclusive, particularly considering different plug types. Broadly speaking, the mating strategy should only apply to polyandrous species, but a sperm protection mechanism matters to all entelegyne spiders. To serve as a sperm protection mechanism, mating plugs should be lodged at spermathecal entrances, while as a mating strategy, mating plugs should present a physical obstacle to sperm entering spermathecae anywhere within the copulatory tract. External examination is oftentimes difficult and reveals very little of whether spermathecal entrances are blocked. Behavioral tests are also deficient as they may reveal that plug presence significantly decreases female remating rates, but cannot at the same time test for their role in sperm protection. Given that mating plugs of both types have in fact been documented to reach spermathecal entrances, e.g., genital plugs in [26–28]; secretion plugs in [8,21], they could plausibly serve both functions at the same time. Whether plugs have originally evolved for one function, then taken the other, is unknown.

We believe that a careful morphological study of the details of inner genitalia might help to clarify these unknowns. The epigynal tracts of a tetragnathid spider *Diphya wulingensis*

provide an ideal model system to test whether mating plugs might serve as a remating prevention, or as a sperm protection mechanism. The spermathecae in *D. wulingensis* have two chambers with the entrance and the exit located oppositely [15]. As many spiders, the blind ball-shaped sac adjacent to copulatory opening in *D. wulingensis* is the secondary spermatheca, while the one linking to fertilization groove is the primary spermatheca [22]. Specially, paired slits connecting between copulatory openings and fertilization grooves extend along the epigynal plate that are accord with the tracts connecting between the two spermathecal chambers beneath the epigynal plate [15]. Such characters imply the spermathecal tracts connecting between the two spermathecal chambers might be in a groove state and the slits are their split openings on the epigynal plate [29], but see [22,30]. If so, one may expect that mating plugs lodged at different sites might have different biological significance: blocking copulatory openings should impede female remating, but to protect sperm from leakage, desiccation and backflow, plugs need not only block copulatory openings but also seal the whole spermathecal groove slits. Furthermore, if mating plugs should serve as a sperm protection mechanism, additional problems would persist. For example, any sperm deposited within the secondary spermathecae would need to first transfer into the primary spermathecae, then be released to fertilization grooves. It is difficult to imagine that mating plugs, which would effectively function for sperm protection, would not being an obstacle for sperm release and female remating.

We first focused on one species to test whether mating plugs serve as sperm protection or a mating strategy, the tetragnathid spider *Diphya wulingensis*. We conducted a morphological

study on epigynal tracts of *D. wulingensis* by scanning electronic microscope (SEM) and semi thin histological series sections (HSS). We untangled the internal structure of epigynal tract and its relationship with the mating plug by 3D-reconstruction based on a set of HSS pictures. If *D. wulingensis* females are polyandrous, we asked how mating plugs can solve the sperm protection issues without hindering female remating, and how, given that the entrance and exit to its secondary spermatheca share a location on a stalk, the sperm leaves it to fertilize eggs. Due to the immense variation in the morphology of entelegyne spider spermathecae, and because various mating plugs may have multiple origins [7], we then undertook a widely comparative study on spermathecal morphology of entelegyne spiders to test whether there are multiple resolutions to sperm protection.

MATERIALS AND METHODS

Adult female *D. wulingensis* were collected from Mt. Wuling (N40.56334º, E117.48740º, alt. 1112−1176m), Beijing, China on 11−12 Aug. 2009. Epigyna of nineteen females were examined, including seventeen by SEM and two for histological study by semi thin HSS. One of the two sets of HSS pictures was used for 3D-reconstruction. For comparative study, epigyna were broadly sampled to maximize phylogenetic representations and to emphasize variation in the anatomy of spermathecae. Besides the spiders examined here, information of some species was collected from the SEM images provided in those previous studies [21,31−38]. To check the state of sperm deposited within the spermathecae, four species, *Neriene emphana* (Linyphiidae), *Parasteatoda tepidariorum* (Theridiidae), *Hahnia zhejiangensis* (Hahniidae)

and *Neoscona* sp. (Araneidae), were also applied for HSS studies. Furthermore, more individuals of *Neriene emphana* (Linyphiidae), *Acanoides hengshanensis* (Linyphiidae) and *Trichonephila clavata* (Nephilidae) were examined to check the occurrence of mating plugs. The collecting data of the materials examined here is provided in S1 Table. All the materials were preserved in 95% ethanol and stored at −80 °C prior to preparation.

Epigyna were primarily examined using a Leica M205A stereomicroscope. To examine epigynal tracts by SEM, seven epigyna of *D. wulingensis* and that of all other species for comparative study, were prepared as described in [39]. Non-chitinous abdominal tissues were digested with Pancreatin (Sigma LP 1750) enzyme complex, then cleared by an ultrasonic cleaner before drying. The other ten epigyna of *D. wulingensis* used for plug examination were only gently cleared with water to avoid mating plug deformation by enzyme and ultrasonic cleaner. SEM images were taken using a LEO 1430VP in the Department of Biological Sciences at George Washington University and a Hitachi S-3400N at China Agricultural University.

The semi-thin serial sections (1μm) were applied to the spider opisthosoma and performed at China Agricultural University using a Leica EM UC6 microtome with a glass knife and stained with toluidine blue (1%) in an aqueous borax solution (1%) at approximately 90°C for 1–4 minutes. All slides were examined using a Leica DM5500B light microscope and images were collected with a Leica DFC 500 camera. Information of epigynal structurers was extracted from the HSS pictures and used for 3D-reconstruction. Data were visualized and processed

using the 3D analysis software Avizo 9.0. The parameters used to build graphs for *D. wulingensis* include: Input resolution (px): 1700*1700*237; Input voxel size: 1*1*2.37; Filter: Lanczos; Mode: dimensions; Resolution (px): 480*480*237; Voxel size: 3.54167*3.54167*2.37. The main points extracted from HSS pictures include epigynal plate, copulatory grooves, fertilization grooves, spermathecae, slits of epigynal tracts, and internal spaces of epigynal tracts. All these structures were lined in color on the black-and-white reversal images in Avizo 9.0.

Abbreviations used in the text, tables and figures:

ACD, paired CD included in an atrium;

ACG, paired CG included in an atrium;

ASCP, single SCP blocking atrium completely;

ASCP-inc, single SCP blocking atrium incompletely;

AT, atrium;

CD, copulatory duct;

CG, copulatory groove;

CGD, CG+CD;

CGDG, CG+CD+CG;

CGF, CG slit on EP fused;

CGSCP, SCP in CG slit;

CGSP, SP in CG slit;

CO, copulatory opening;

COGP, GP in CO;

COSCP, SCP in CO;

COSP, SP in CO;

CPG, pocket-like CG;

CT, copulatory tract;

EP, epigynal plate;

EPSCP, SCP on EP coving over both SG slits and CO;

EPSP, SP on EP;

ET, epigynal tract;

FD, fertilization duct;

FD+CFD, paired FD convergent into common FD;

FDG, FD+FG;

FDG+CFG, paired FD+FG convergent into common FG;

FG, fertilization groove;

FG+AFD, FG with additional FD;

FG-OEF, FG slit ending outside epigastric furrow;

FT, fertilization tract;

GC, groove cavity;

GE, groove edge;

GP, genital plug;

HCO, CO hidden;

S, spermatheca;

S_1, primary spermatheca;

S_2, secondary spermatheca;

Scape, EP protruding and prolonged, carrying CG out, CO located at distal part of scape.

SCD, spermatheca of compacted duct;

SCG, spermatheca of compacted groove;

SCP, secretion plug;

SD, spermathecal duct;

SED, spermatheca of elongated duct;

SEG, spermatheca of elongated groove.

SG, spermathecal groove;

SGF, SG slit on EP fused;

SGSCP, SCP in SG slit;

SGSP, SP in SG slit;

SP, sperm plug;

SP+SCP, SP and SCP coexist;

SSCP, SCP at spermathecal entrance.

ST, spermathecal tract

RESULTS

In *D. wulingensis* the epigynum consists of a plate located on the abdomen surface in front of the epigastric furrow, and of a pair of epigynal tracts under it (Fig 1A–D). The epigynal plate is sclerotized, slightly concaved centrally, and bulged posteriorly. The copulatory openings are located in the centrally concaved area. A pair of slits, starting from the copulatory openings, extends vertically along the epigynal plate and anteriorly into the epigastric furrow. The paired epigynal tracts beneath the integument, consisting of the copulatory tract (CT), the fertilization tract (FT), and the spermatheca (S), accord with a general model of CT-S-FT. The spermathecae in many spiders are globular, but in *D. wulingensis* they are elongate and tubular, and thus resemble tracts, with their entrance and exit located at two ends (Fig 1D). Each spermatheca consists of two spermathecal chambers: a U-shaped tract (S_1) and a blind ball-shaped sac (S_2), as well as a spermathecal tract (ST) connecting them. The two spermathecal chambers, S_1 and S_2, traditionally termed as primary and secondary spermathecae [22], possess numerous gland pores on the wall (Fig 1E), and link the fertilization with the copulatory tract, respectively (a

tract model of CT-S_2-ST-S_1-FT). Considering the proximity of the paired copulatory openings with the S_2 entrances, the CT in this species is extremely short. The paired FT in the inner, dorsal side of the epigynum, connects the S_1 and extends convergently into the epigastric furrow.

Fig 1. Epigynum and mating plugs of *Diphya wulingensis*. A, ventral view, arrow points to fragmented SP in SG; B, caudal view, C, dorsal view; D, inner view, arrows indicate direction of sperm flow; E, detail of D, shows gland pores on S_1 and S_2; F–H, ventral view, show mating plugs on epigynal plate, arrow in H points to SP spilled from SG. CG, copulatory groove; CO, copulatory opening; FG, fertilization groove; S_1, primary spermatheca; S_2, secondary spermatheca; SCP, secretion plug; SG, spermathecal groove; SP, sperm plug. Scale bars: mm.

Epigynal tracts are integrated with the epigynal plate in *D. wulingensis*. The inner view shows that the tracts are not separated from the epigynal plate (Fig 1D). The cross sections of the epigynal tracts are not closed circles, but have a break opening on epigynal surface, as the integument of epigynal plate continues with the inner surface of the tracts (Fig 2). The tract wall in cross sections is specially modified into a tadpole-shape (Fig 2D–E), the bottom cavity open on the epigynal surface through the "tail" part. Comparatively, the walls of copulatory tracts and spermathecae are as thick and strongly sclerotized as the integument of the epigynal plate, while the walls of fertilization tracts are much thinner, less sclerotized (Fig 2F–H), and are easily broken during specimen preparation (Fig 1D). In addition, the two chambers and the duct between them in *Hahnia zhejiangensis* (Hahniidae) have sperm inside (S1 Fig D–E) that indicate both of them are spermathecal chambers, and the duct between them are spermathecal ducts, rather than copulatory ducts as usually recognized (but see [40]). No evidence shows that the sperm deposited in the spermathecae would be encapsulated in discrete packages in order

to avoid the mixing of sperm from different males (Fig 2C, see also S1 Fig) as reported in some spiders [41].

Fig 2. Cross sections of epigynum of *Diphya wulingensis*. A, 3D-reconstructed epigynum, ventral view; B, internal space of reconstructed tracts; lines in A–B show section positions of C–H; C, section cross CG; D, section cross S_2 stalk, left connecting to CG, right to SG; E, section cross SG; F, section cross SG and S_1; G, section cross S_1 and FG; H, section cross FG. CG, copulatory groove; DF, dorsal fold; FG, fertilization groove; S_1, primary spermatheca; S_2, secondary spermatheca; SCP, secretion plug; SG, spermathecal groove.

The 3D-reconstructed epigynal tracts in *D. wulingensis* are “grooves”, rather than closed “ducts” (a tract model of CG-S_2-SG-S_1-FG, hereafter). In the reconstructed model, the groove bottom cavity and the “tail” part, extend through the entire epigynal tract (Fig 3), the “tail” parts join together to form an edge of the grooves, and the “tail” end openings connect to become a pair of slits on the epigynal plate. From the reconstructed model one can see that the structure of the spermathecal groove (SG) consists of groove cavity (GC) and groove edge (GE), the two spermathecal chambers: S_1 and S_2 are derived from the groove cavity (Fig 4). As a U-shaped tract, S_1 is formed by a part of groove cavity enlarged into a pocket, with the two integument layers tightly close to each other at the central area (Fig 4A), and laterally folded (Figs 2E), resulting in a U-shaped chamber along the pocket bottom (Figs 1D, 3H, 4A), with one end linking to the fertilization groove (Fig 3F). The blind ball-shaped S_2 arises from the distal end of the spermathecal groove cavity and resembles a cul-de-sac spermatheca, having the entrance and exit included within the same basic stalk (Fig 1D). However, the inner space of the stalk is also modified into a tadpole-shaped in cross section, having the “tail” part linked to the copulatory groove and the cavity part serving as an exit to the spermathecal groove (Figs 3H,

3I, 4A, 4C). This makes the incoming and outflowing sperm do not equally share its internal space. Structurally, the S_2 stalk not only docks to the spermathecal groove, but also has its edge in part extend distally to the copulatory opening. Therefore, the copulatory groove is comprised only of the edge part, without a bottom cavity (Figs 3I, 4B), and is extremely short, making the entrances of S_2 adjacent to the copulatory openings (Fig 3H). The slits of fertilization grooves open on the epigynal dorsal surface (Fig 3C). Consequently, the paired slits on the epigynal plate (Fig 1A) match the slits of spermathecal grooves connecting between S_1 and S_2 (Fig 3A, 3I). Besides spermathecal entrances, the groove-shaped spermathecae in *D. wulingensis* also have paired slits, opening on the epigynal plate. Although we did not find any internal structures that might close the spermathecal entrances and groove slits, the modified spermathecal walls prevent the groove cavities to be directly opened on the epigynal plate, but rather via the groove edges which have the two layers tightly closed (Figs 3I, 4).

Fig 3. 3D-reconstructed epigynum of *Diphya wulingensis*. A, C, E, G, epigynal plate and epigynal tracts, with integument of epigynal plate and tract wall semitransparent, those in thumbnails opaque; B, D, F, H, I, internal space of epigynal tracts; A–B, ventral view; C–D, caudal view; E–F, dorsal view; G–H, inner view, arrows in H indicate direction of sperm flow, blue line shows cross section of S_2 stalk; I, lateral view, red line indicates slit opening on epigynal plate. CG, copulatory groove; FG, fertilization groove; GC, groove chamber; GE, groove edge; S_1, primary spermatheca; S_2, secondary spermatheca; SG, spermathecal groove.

Fig 4. Schematic structure of epigynal tract of *D. wulingensis*. A, schematic tract, space represents spermathecal chamber and groove cavity, shadow represents groove edge, double lines indicate groove slit on epigynal plate, blue lines indicate section positions in B–D; B, section cross CG and S_2; C, cross section of S_2 stalk; D, cross section of SG. CG, copulatory groove; CO, copulatory opening; FG, fertilization groove; GC, groove cavity; GE, groove edge; S_1, primary spermatheca; S_2, secondary spermatheca; SG, spermathecal groove.

The amorphous plugs found in *D. wulingensis* vary in size and texture (Fig 1F–H). The different textures suggest that plug materials are different in their density and liquidity. Two types can be recognized: sperm plug (hereafter SP, Fig 1F) and secretion plug (hereafter SCP, Fig 1G). The SP materials usually contain many rounded sperm-like pellets, nesting within groove slits or flattened on the central concaved area of the epigynal plate, intact or fragmented (Fig 1F, 1H). The hardened scabs are thin and fragile, usually have a flat or slightly concaved surface that indicates their plug materials are thin liquids with good fluidity. The hardened SCP materials have a bumpy surface and are difficult to be removed by dissection without destroying the epigynal tracts (Fig 1G). These indicate the SCP materials are much denser and harder than those of SP. Some SCP materials also contain rounded sperm-like pellets, but when present, these are covered under thick secretions. This suggests that SCP are added upon the existing SP (Fig 1H). In HSS pictures the SCP materials are different from the sperm deposited within the spermathecae in color and texture, and are separated from the sperm in the groove cavity by the groove edge (Fig 2). In some cases, the entire ventral surface of the epigynal plate can be covered with the SCP, including the central concaved area, as well as the posterior bulged area (Fig 1G). In these cases, the copulatory openings and the paired spermathecal groove slits on the ventral surface are overlaid entirely. In some cases, the SCP is less complete with the plug materials only covering a part of the groove slits, or the slit of one side only (Fig 1H). In all these cases, plug materials are only found on the ventral epigynal surface, leaving fertilization groove slits on the dorsal surface free.

Among the 17 epigyna of *D. wulingensis* examined by SEM, five had SP at both sides, fragmented or intact; eight had a complete SCP simultaneously sealing copulatory openings and the slits on the ventral surface, four had a SP on one side and SCP on the other, and in two cases the incomplete SCP did not block the entire groove slits (Table 1; Fig 1). Both SP and SCP remained intact after being treated by enzymes. However, in one case, after having the epigynum treated by both enzymes and supersonic cleaner, the sperm plug became fragmented with small pieces nested within groove slits, and in another case the large SP scab was broken by tweezers during mounting.

Table 1. Mating plugs in *Diphya wulingensis*

Specimen	Enz	SC	Plug type	Notes
No. 01			SP-SP+SCP	incomplete SCP
No. 02			SCP	complete SCP
No. 03			SP	intact
No. 04			SP	intact
No. 05			SCP	complete SCP
No. 06			SP	broken
No. 07			SCP	complete SCP
No. 08			SP-SCP	incomplete SCP
No. 09			SCP	complete SCP
No. 10			SP-SCP	incomplete SCP
No. 11	+		SP-SCP	incomplete SCP
No. 12	+		SP	intact
No. 13	+		SCP	complete SCP
No. 14	+		SCP	complete SCP
No. 15	+	+	SP	small pieces
No. 16	+	+	SCP	complete SCP
No. 17	+	+	SCP	complete SCP

Enz, epigyna treated by enzymolysis; SC, epigyna treated by supersonic cleaner; SP, sperm plug; SCP, secretion plug; SP+SCP, SP and SCP coexist; SP-SCP, SP at one side, SCP at the other side.

Besides *D. wulingensis*, additional 394 epigyna from 330 species, 187 genera, 36 families were also examined here. Based on the locations of the two openings and the architecture of spermathecae, we divided these entelegyne spermathecae into four types (Fig 5; Table 2): The first broad distinction is between tract-shaped spermathecae with the entrance and exit located at each end, or sac-shaped spermathecae having the entrance and exit closed. The tract-shaped spermathecae usually have two chambers, S_1 and S_2, at each end, the tract connecting between them also has a slit opening on the epigynal plate, like that in *D. wulingensis* (Fig 1D), we term this type as spermathecae of elongated groove (SEG, a tract model of CG-S_2-SG-S_1-FG, Fig 5A–B). Such elongated spermathecae may have the slits of spermathecal grooves partly or totally fused, which renders the spermathecal grooves into closed ducts; we term this type as spermathecae of elongated duct (SED, a tract model of CD-S_2-SD-S_1-FG, Figs 5C–D, 6). In some cases, the closed ducts are separated from the integument (Fig 5E–F). Those sac-shaped spermathecae may arise from the groove bottom cavity with the entrance and exit close to each other, and we term this condition as spermathecae of compacted groove (SCG, a tract model of CG-S-FG, Fig 5G–H, or CD-S-FG, Table 2). Alternatively, those spermathecae usually have one sac-shaped chamber, separated from the groove/duct, and instead have a pair of ducts, CD and FD connected to the epigynal tract; we term this condition as spermathecae of compacted duct (SCD, a tract model of CD-S-FD, Fig 5I–J). Most species examined in this study have the spermathecae of SCG type due to the dense sampling of linyphiids, the spermathecae of the other three types are also common among other spider groups (S2 Table). Variation can be found in some spiders, such as one of the two spermathecal chambers reduced or lost (Fig 6), as well as copulatory and fertilization tracts transform between groove and duct.

Table 2. Spermathecal types in entelegyne spiders

S-type	ET-style	Species name	Figure	ET characters	Plug on EP
SEG	CG-SEG-FG	*Diphya wulingensis*	Fig 1	tract-shaped spermathecae with paired SG slits on EP	EPSCP/EPSP
SEG	CG-SEG-FG	*Tamgrinia alveolifera*	Fig 5A,5B,5K	tract-shaped spermathecae with paired SG slits on EP	SGSP
SED	CD-SED-FD+CFD	*Hahnia zhejiangensis*	Fig 5E,5F,5M	tract-shaped spermathecae having paired CO on EP	—
SED	CD-SED-FG	*Leucauge* sp.	Fig 5C,5D,5L	tract-shaped spermathecae with fusing traces of SG slits on EP	—
SCG	CG-SCG-FG	*Neriene emphana*	Fig 5G,5H,5N	paired CG slits on ventral side of EP and paired FG slits on dorsal side	CGSCP
SCD	CGD-SCD-FD+CFD	*Parasteatoda tepidariorum*	Zhan et al. 2019: Fig1E–1G	sac-shaped spermatheca having CD and FD for sperm income and outcome respectively, paired FD convergent into CFD	—
SCD	CGD-SCD-FDG+CFG	*Trichonephila clavata*	Fig 5I,5J,5O	sac-shaped spermatheca having CD and FD connecting to CG and FG respectively, paired FG convergent into CFD	CGSP/COSCP

Epigynal elements: CD, copulatory duct; CG copulatory groove; CGD, CG+CD; CO, copulatory opening; EP, epigynal plate; ET, epigynal tract; FD, fertilization duct; FG, fertilization groove; FDG, FD+FG; CFD, common FD; S, spermatheca; SG, spermathecal groove.

S-type: SCD, spermathecae of compacted duct; SCG, spermathecae of compacted groove; SED, spermathecae of elongated duct; SEG, spermathecae of elongated groove.

Mating plug type: SCP, secretion plug; SP, sperm plug.

Plug on EP, mating plug lodged on epigynal plate; COSCP, SCP in CO; CGSCP, SCP in CG slits; CGSP, SP in CG slit; EPSCP, single SCP on EP coving over both CO and SG slits; EPSP, SP on EP; SGSP, SP in SG slit.

Fig 5. Types of entelegyne spermathecae. A, B, K, Type-I SEG, arrow in A points to SP in SG slit; C, D, L, Type-II SED, arrow in C points to fusing trace of SG slit on EP; E, F, M, Type-II SED, without fusing trace on EP; G, H, N, Type-III SCG; I, J, O, Type-IV SCD. A–B, *Tamgrinia alveolifen* (Agelenidae); C–D, *Leucauge* sp. (Tetragnathidae); E–F, *Hahnia zhejiangensis* (Hahniidae); G–H, *Neriene* sp. (Linyphiidae); I–J, *Trichonephila clavate* (Nephilidae); A, C, E, G, I, epigynum ventral view; B, D, F, H, J, same, inner view; K–O, schematic drawings of four spermathecal types. AT, atrium; CD, copulatory duct; CF, common FD; CG, copulatory groove; CO, copulatory opening; FD, fertilization duct; FG, fertilization groove; EP, epigynal plate; S, spermatheca; S_1, primary spermatheca; S_2, secondary spermatheca; SCD, spermatheca of compacted duct; SCG, spermatheca of compacted groove; SD, spermathecal duct; SED, spermatheca of elongated duct; SEG, spermatheca of elongated groove; SG, spermathecal groove; SP, sperm plug. Scale bars: mm.

Fig 6. Variation in elongated spermathecae of entelegyne spiders. A–B, *Nomisia ausserer* (Gnaphosidae), shows S_2 without significant lumen, arrow points to fusing trace of SG slit; C–D, *Metleucauge yunohamensis* (Tetragnathidae), shows S_1 lost, S_2 as the only significant receptacle; E–F, *Kishidaia albimaculata* (Gnaphosidae), shows well developed S_1 and small S_2, arrow points to fusing traces of SG slits. CO, copulatory opening; FG, fertilization groove; S_1, primary spermatheca; S_2, secondary spermatheca; SCP, secretion plug; SD, spermathecal duct; SG, spermathecal groove; SP, sperm plug. Scale bars: mm.

Mating plugs were checked out from137 of the 411 materials examined here, and can be found in all the four types of epigyna (S2 Table). In contrast to genital plug (GP), which was only found in six materials, SP and SCP are more common. In most cases, plug materials were found in the copulatory openings (Figs 6E, 7F) and the copulatory groove slits (Figs 5A, 5I, 7E, 7I), or in the atria that include both copulatory openings. Furthermore, in those taxa with SEG spermathecae and those with SED spermathecae having their spermathecal groove slits partly fused, plug materials also seal the spermathecal groove slits (Figs 5A, 6A), even in some cases occupy a large area of the epigynal plate (Figs 1G, 6C). In some spiders, more than one type mating plugs coexist (Fig 7), and in some other cases, the plugs of same type may have different plugging states (Figs 1G–H; 7J–K). In those having both SP and SCP can be found (Fig 7), comparatively, SP materials look like having been spilled out from the copulatory openings or groove slits, but do not necessary block them (Figs 1F, 5I, 7H), while those of SCP usually seem to be lodged externally (Figs 1G–H, 6C, 7C, 7F). However, SCP might have different blocking targets. Unlike that in *D. wulingensis*, SCP in *T. clavata* and *A. hengshanensis* focus mainly on the copulatory openings, as well as in part on the copulatory groove slits (Fig 7C, 7F), but in *N. emphana*, plug materials extend along the copulatory groove slits from the copulatory openings to the spermathecal entrances. These plugs aim to block the spermathecal entrances (Fig 7I).

Fig 7. Mating plugs in entelegyne spiders. A−C, *Trichonephila clavata* (Nephilidae), show SP in A−B, SCP in C; D−F, *Acanoides hengshanensis* (Linyphiidae), show SP in D−E, SCP in F; G−I, *Neriene emphana* (Linyphiidae), show SP in G−H, SCP in I; J−K, *Agelena sylvatica* (Agelenidae), show SCP blocking incompletely in J and completely in K; L, *Araneus diadematoides* (Araneidae), shows GP and SP coexist. AT, atrium; CO, copulatory opening; GP, genital plug; SCP, secretion plug; SP, sperm plug. Scale bars: mm.

DISCUSSION

Entelegyne spiders have to deal with the problems of sperm protection caused by the inherent imperfections of the female spermathecal structure: potential leakage and desiccation of sperm after copulation, and potential backflow when sperm are released. Given the immense diversity of spider forms (50 thousand species), these problems might have been resolved in different evolutionary ways. We show that *D. wulingensis* possesses groove-shaped spermathecae (SEG type) having a paired slit-like openings on the epigynal plate and two chambers, S_1 and S_2 located at each end. The base of S_2 has its entrance and exit within the same stalk and close to the copulatory opening, and S_1 links to the fertilization groove. Such structural characteristics require a corresponding sperm protection mechanism in the shape of an amorphous plug, not only to block the spermathecal entrances, but also to seal the spermathecal slits. Furthermore, these plugs would not hinder the release of sperm from S_2, and would not impede the female's subsequent mating. We found two types of mating plugs in *D. wulingensis*, and their different texture and morphology allow us to label them as sperm, and as secretion plugs, respectively. Our results suggest that a complete secretion plug might serve as a permanent protection mechanism in postcopulation, while the modified spermathecal wall together with the spilled sperm form a temporary protection mechanism to prevent sperm from leakage and desiccation intermating. Furthermore, with the modified spermathecal wall of S_2 stalk, the problems of shunt for sperm input and output have been resolved, while at the same time allowing for female polyandrous mating possibilities.

The reconstructed spermathecae of *D. wulingensis* show that a sperm protection mechanism is necessary. As in many entelegyne spiders having the SEG type spermathecae, this spermatheca consists of two chambers, S_1 and S_2, associating with the fertilization tract and the copulatory tract, respectively (Fig 3). Our results show that the epigynal tracts are not separated from the epigynal plate (Fig 1D), their cross sections are not closed circle (Fig 2), and the breaks in the reconstructed model connect to become a pair of slits on the epigynal plate (Fig 3). These indicate that the epigynal tracts are in a groove state [29], following a model CG-S_2-SG-S_1-FG. The paired slits on the epigynal plate (Fig 1A) are not superficial sutures as previously recognized [22,30], but are rather slit-openings of the epigynal grooves. We have not found any internal structures that might close the spermathecal opening and groove slits (Figs 2–3). Similar circumstance can also be found in *Stegodyphus* spiders (Eresidae) [38]. Given that the copulatory grooves are extremely short, and that the fertilization grooves open on the dorsal surface, the paired slits on the ventral surface are largely the openings of the spermathecal grooves. Their walls are thick and strongly sclerotized without any internal closing mechanism to prevent sperm leakage. The fact that SP is found in many other spiders (Figs 5I, 7; S2 Table) indicates that sperm leakage is a common problem in spiders. In *D. wulingensis* both SCP that covers the entire epigynal plate, and SP nested within the spermathecal slits, are in line with the prediction of the sperm protection hypothesis.

A complete SCP might serve as a long-lasting mechanism for sperm protection. Given the extremely short copulatory grooves in *D. wulingensis*, the SCP plugs lodged in copulatory openings also block the spermathecal entrances (Fig 1D). That a complete SCP covering both

the copulatory openings and spermathecal slits (Fig 1G) indicates its function beyond merely impeding female remating. Furthermore, formed by thick secretions, SCP is solid and durable, and is difficult to be experimentally removed. These characteristics suggest that SCP meets the expectations of a sperm protection mechanism that can function permanently. By forming a physical barrier to sperm insemination at the same time, SCP does, however, also lower the possibility of subsequent mating of the female. The texture of the incomplete SCP reveals a thick and well solidified material whose external formation on the epigynal plate (Fig 1H) cannot be accidental, but suggests it be rather intentional. However, our data show that not every mating results in SCP formation (Table 1): the common presences of mating plugs, either SP or SCP, indicate that all the females examined had mated; eight of them had a complete SCP (8/17), while the others had an incomplete SCP or only a SP. This suggests that the polyandrous mating system in *D. wulingensis* is not jeopardized by plug formation. It thus seems that SCP is responsible for postcopulatory sperm protection.

Our data imply that the modified wall of spermathecal groove together with the spilled sperm form a temporary sperm protection mechanism in *D. wulingensis*. While grooved epigynal tracts have been reported in some entelegyne spiders [22,29,42–46], most of them are relevant to the groove-shaped copulatory and fertilization tracts. Nevertheless, *D. wulingensis*, as well as those spiders also having the SEG spermathecae, have also grooved spermathecae (Figs 1A, 5A; see also [38]: figs 1–3; [47]: figs 38–41; S2 Table). As stated above, potential sperm spills from the slits, be it post copulation or intermating, would not be surprising given the vagaries of the entelegyne system. Spilled sperm liquid captured between the two edge

layers of the specifically modified spermathecal wall (Figs 2E, 3I, 4D) may solidify under surface tension thereby providing a temporary seal of the spermathecal slits. We hypothesize that this mechanism prevents the sperm deposited in the spermathecae from leakage and desiccation. We thus conclude that SP in *D. wulingensis* serves as a temporarily sperm protection mechanism during the intermating stage. Similar SP can also be found in other species with the spermathecae of the SEG type (Fig 5A).

These mating plugs, be it SP or SCP, do not prevent sperm to be released from S_2 and an existing SP does not constitute an obstacle to the mating of subsequent males. Generally, SP is formed by the spilled sperm liquid left between the two layers of the groove edge that has little effects to the sperm flow through the groove cavity (Fig 4A). The SCP, on the other hand, is formed by the hardened secretions pasted externally onto the epigynal plate (Fig 1G–H), although we have no information about where the secretions come from. HSS analyses reveal that the plug material has a different texture from the sperm stored in S_1, S_2 and the spermathecal groove (Fig 2). This suggests that separated by the edge part, the thick secretions cannot get into the spermathecal cavities via the spermathecal slits. Nevertheless, S_2 is a blind sac arising from the groove cavity, and has its entrance and exit share the same stalk (Fig 1D); hence, sperm inflow and outflow must share the same way. It would thus be challenging to install a mechanism only to prevent sperm leakage, and not also sperm released. The reconstructed epigynal tracts show that the internal space of S_2 stalk is modified into a tadpole-shape in cross section (Figs 2D–E, 4C), having the wide cavity part dock to the cavity of spermathecal groove, while the narrow edge part associates with the edge of the spermathecal groove, and extends

distally to the copulatory opening, forming the copulatory groove with only the edge part, without a cavity (Figs 3H, 3I, 4B). This makes sperm intake to, and sperm outflow from S_2 differ fundamentally. During mating the male uses its pointed embolus (see figures in [48]) to penetrate the edge of the copulatory groove and to inject sperm directly into the S_2. Although sperm liquid may spill out as the embolus is withdrawn, this mechanism facilitates the spilled sperm and the modified spermathecal wall to form a SP sealing, thereby effectively preventing sperm leakage and desiccation. This has little effect on sperm release from S_2 to S_1 through the spermathecal groove, and further to the secondary uterus externus [15]. Furthermore, being a thin and fragile layer, such a SP does not prevent the embolic insertion of a subsequent male [49]: it therefore does not prevent female polyandry.

Elongated spermathecae of SEG or SED types are common in many entelegyne spiders (Fig 5A–F, see also [22,38,47]. Ontogenetic evidence suggests that the development of epigynal tracts in entelegyne spiders starts with a pair of teguments folds (grooves) including S_1 and S_2 (S_1= ‘‘base of spermatheca,’’ and S_2 (= ‘‘head of spermatheca,’’ [30]). This suggests that the elongated spermatheca of the SEG type represents a plesiomorphic condition, while other types are derived. Furthermore, an ‘‘accessory bulb’’ of [50,51] is homologous to the ‘‘head of spermatheca’’ [22,30], and the ducts between S_1 and S_2 in *Hahnia zhejiangensis* are in fact the spermathecal tracts in a duct state (Fig 5E–F), rather than copulatory ducts as previously recognized [40]. The HSS evidence also shows that both S_1 and S_2, as well as the ducts between them, have sperm deposited inside (S1 Fig D–E). Our presented comparative evidence shows that besides the modification of the spermathecal wall in *D. wulingensi*s, further variation in

spermathecal grooves includes: 1) having the groove slits fused (SED type, Fig 5C–D); 2) the fused spermathecal grooves further becoming internal ducts (SED type, Fig 5E–F); 3) the tracts becoming shortened that let the openings and exits close to each other (SCG type, Fig 5G–H); 4) having one of the chambers reduced or even lost (Fig 6); 5) becoming sac-shaped spermathecae, having CD and FD associating to CG and FG respectively (Figs 5I–J, 6). All these changes are likely adaptations to resolving the problem of sperm spilling out from the slits of spermathecal grooves.

Considering mating plugs of varying shapes and origins in the vast morphospace of spiders—a hyper-diverse animal group—, we certainly allow for the possibility that different plugs serve different purposes, and we do find more than one type mating plug coexist in some spiders (Fig 7, see also [12]. These can be adaptations for mating strategies, as well as for sperm protection. Besides those plugs that are formed by parts of broken-off male genital parts [7,27,28], SCP may also well relate only to mating strategies (Fig 6C, 6F, 6I), since they only block the copulatory openings and parts of the copulatory groove slits. However, we suggest that amorphous plugs have evolved for sperm protection, and not to prevent female polyandry. If the presence of irremovable mating plugs presents a physical barrier to female remating, then perhaps that is its secondary function. In fact, all entelegyne spiders require a means of sperm protection due to the inherent defects of such spermathecal morphology, while many spiders do not engage in polyandrous mating that may give credibility to this explanation. Therefore, in the species where mating plugs do function as a mating strategy [7,12], future empirical

studies should also test for their sperm protection function, and comparative studies might eventually learn what function evolved first.

Conclusions

As we demonstrate above with detailed morphological comparations, all entelegyne spiders have to deal with the problems of sperm protection caused by the inherent imperfections of the female spermathecal structure[illegible] In *D. wulingensis*, having SEG type spermathecae and female polyandry, the specially modified spermathecal groove wall together with the spilled sperm form SP to function as a temporary protection mechanism to prevent sperm from leaking and desiccating, the externally lodged SCP functions in postcopulation both to prevent additional matings, and serve as a permanent sperm protection mechanism. Furthermore, with the modified spermathecal wall of S_2 stalk, the problem of shunt for sperm input and output has been resolved, and the SP would not impede the subsequent matings of the female. Similar circumstances that more than one type of mating plugs present in the same spider also can be in some other spider groups (Fig 6, see also [12]). Our results suggest that mating plugs might not only serve as mating strategy in spiders, as having been proved previously, but also as a sperm protection mechanism; different types of mating plugs might function in different ways; and the problem of sperm protection in the spiders of different type of spermathecae might have multiple resolutions. The present study provides the first empirical evidence for the hypothesis of mating plug serving as a sperm protection mechanism. This is attributed to the special morphological features of the SEG type spermathecae in *D. wulingensis*. More often in spiders

it is difficult to distinguish the original purpose of an exist mating plugs whether is to prevent female's remating, or to protect the sperm. Considering the multiple and unknown origins of mating plugs in spiders, in future, detailed study on epigynal morphology and copulation behavior trial to show the formation of mating plugs would help to get more evidence for the sperm protection hypothesis.

Author contributions

Conceptualization: HJ, MK, LT.

Data curation: HJ, YZ, QW, LT.

Formal analysis: HJ, YZ, QW.

Funding acquisition: HZ, LT.

Investigation: HJ, YZ, LT.

Methodology: HZ.

Software: HZ.

Visualization: HJ.

Writing – original draft: LT.

Writing – review & editing: HJ, MK, LT.

Acknowledgements

We would like to thank Jinzhong Fu for his comments on an earlier version of this paper. We also thank Fernando Álvarez-Padilla, Lara Lopardo, Dimitar Dimitrov, and Gustavo Hormiga for their help on collection for SEM images, and Feng Zhang and Yuri M. Marusik for their

help in spiders' identifications. We thank these colleagues who facilitated institutional loans: Norman Platnick and Louis Sorkin (American Museum of Natural History), Shuqiang Li (Institute of Zoology, Chinese Academy of Sciences), Yuri M. Marusik (Institute for Biological Problems of the North, Russian Academy of Sciences), Seppo Koponen (Zoological Museum, University of Turku), Andrei Tanasevitch (Institute of Ecology and Evolution, Russian Academy of Sciences), Hirotsugu Ono (Department of Zoology, National Science Museum, Tokyo) and Akio Tanikawa (School of Agriculture and Life Sciences, University of Tokyo).

References

1. Barker DM. Copulatory plugs and paternity assurance in the nematode *Caenorhabditis elegans*. Vol. 48, Animal Behaviour. 1994. p. 147–56.
2. Polak M, Wolf LL, Starmer WWT, Barker JSF. Function of the mating plug in *Drosophila hibisci* Bock. Behav Ecol Sociobiol. 2001;49(2–3):196–205.
3. Sauter A, Brown MJF, Baer B, Schmid-Hempel P. Males of social insects can prevent queens from multiple mating. Proc R Soc B Biol Sci. 2001;268(1475):1449–54.
4. Contreras-Garduño J, Peretti A V., Córdoba-Aguilar A. Evidence that mating plug is related to null female mating activity in the scorpion *Vaejovis punctatus*. Ethology. 2006;112(2):152–63.
5. Timmermeyer N, Gerlach T, Guempel C, Knoche J, Pfann JF, Schliessmann D, et al. The function of copulatory plugs in *Caenorhabditis remanei*: Hints for female benefits. Front Zool. 2010;7(1):28.
6. Helsdingen PJ van. Sexual behaviour of *Lepthyphantes leprosus* (Ohlert) (Araneida, Linyphiidae), with notes on the function of the genital organs. Zool Meded. 1965;41:15–42.
7. Uhl G, Nessler SH, Schneider JM. Securing paternity in spiders? A review on occurrence and effects of mating plugs and male genital mutilation. Genetica. 2010;138(1):75–104.
8. Sentenská L, Pekár S, Lipke E, Michalik P, Uhl G. Female control of mate plugging in a female-cannibalistic spider (*Micaria sociabilis*). BMC Evol Biol. 2015;15(18):1–12.
9. Schneider JM, Thomas ML, Elgar MA. Ectomised conductors in the golden orb-web spider, *Nephila plumipes* (Araneoidea): A male adaptation to sexual conflict? Behav Ecol Sociobiol. 2001;49(5):410–5.
10. Nessler SH, Uhl G, Schneider JM. Genital damage in the orb-web spider *Argiope bruennichi* (Araneae: Araneidae) increases paternity success. Behav Ecol. 2007;18(1):174–81.
11. Foellmer MW. Broken genitals function as mating plugs and affect sex ratios in the orb-web spider *Argiope aurantia*. Evol Ecol Res. 2008;10(3):449–62.
12. Kuntner M, Gregorič M, Zhang S, Kralj-Fišer S, Li D. Mating plugs in polyandrous giants: Which sex produces them, when, how and why? PLoS One. 2012;(7).
13. Huber BA. The retrolateral tibial apophysis in spiders-shaped by sexual selection? Zool J Linn Soc. 1995;113(2):151–63.
14. Foelix R. F. Biology of spiders. Third Edit. Oxford University Press. Oxford University Press; 2011. 428 p.
15. Zhan Y, Jiang H, Wu Q, Zhang H, Bai Z, Id LT. Comparative morphology refines the conventional model of spider reproduction. PLoS One. 2019;14(7):e0218486.
16. Fromhage L, Schneider JM. Emasculation to plug up females: The significance of pedipalp damage in *Nephila fenestrata*. Behav Ecol. 2006;17(3):353–7.
17. Uhl G, Busch M. Securing paternity: Mating plugs in the dwarf spider *Oedothorax retusus* (Araneae: Erigoninae). Biol J Linn Soc. 2009;96(3):574–83.
18. Eberhard WG, Huber BA. Spider Genitalia. In: Leonard JL, Córdoba-Aguilar A (eds) The evolution of primary sexual characters in animals. Leonard J, Córdoba-Aguilar A, editors. The Evolution of Primary Sexual Characters in Animals. Oxford University Press, New York; 2010. 249–284 p.
19. Knoflach B. Mating in *Theridion varians* Hahn and related species (Araneae: Theridiidae). J Nat Hist. 1998;32(4):545–604.
20. Knoflach B. Diversity in the copulatory behaviour of comb-footed spiders (Araneae, Theridiidae). In: Thaler, K. (ed.) Diversität und Biologie von Webspinnen, Skorpionen und anderen Spinnentieren. Denisia. 2004;12:161–256.
21. Álvarez-Padilla F, Hormiga G. Morphological and phylogenetic atlas of the orb-weaving spider family Tetragnathidae (Araneae: Araneoidea). Zool J Linn Soc. 2011;162(4):713–879.

22. Ramírez M. The morphology and phylogeny of dionychan spiders (Araneae: Araneomorphae). Bull Am Museum Nat Hist. 2014;390:1–374.
23. Jackson RR. The mating strategy of *Phidippus johnsoni* (Araneae, Salticidae): II. Sperm competition and the function of copulation. J Arachnol. 1980;8(3):217–40.
24. Masumoto T. The Effect of the Copulatory Plug in the Funnel-Web Spider, *Agelena limbata* (Araneae: Agelenidae). Ecol Res. 1993;21:55–59.
25. Helsdingen PJ van. A reclassification of the species of *Linyphia* Latreille based on the functioning of the genitalia (Araneida, Linyphiidae), Part I. *Linyphia* Latreille and *Neriene* Blackwall). Zool Meded. 1969;1–302.
26. Berendonck B, Greven H. Morphology of female and male genitalia of *Latrodectus revivensis* Shulov, 1948 (Araneae, Theridiidae) with regard to sperm priority patterns. In: Toft, S & N Scharff (eds) European Arachnology 2000: Proceedings of the 19th European Colloquium of Arachnology Aarhus University Press. 2002. 157–67 p.
27. Kuntner M, Kralj-Fišer S, Schneider JM, Li D. Mate plugging via genital mutilation in nephilid spiders: An evolutionary hypothesis. J Zool. 2009;277(4):257–66.
28. Kuntner M, Coddington JA, Schneider JM. Intersexual arms race? Genital coevolution in nephilid spiders (Araneae, Nephilidae). Evolution (N Y). 2009;63(6):1451–63.
29. Tu L, Hormiga G. The female genitalic morphology of "micronetine" spiders (Araneae, Linyphiidae). Genetica. 2010;138(1):59–73.
30. Sierwald P. Morphology and ontogeny of female copulatory organs in American Pisauridae, with special reference to homologous features (Arachnida, Araneae). Smithson Contrib to Zool. 1989;484:1–24.
31. Dimitrov D, Benjamin SP, Hormiga G. A revised phylogenetic analysis for the spider genus *Clitaetra* Simon, 1889 (Araneae, Araneoidea, Nephilidae) with the first description of the male of the Sri Lankan species *Clitaetra thisbe* Simon, 1903. Bull Museum Comp Zool. 2009;159(6):301–23.
32. Griswold CE, Ramírez MJ, Coddington JA, Platnick NI. Atlas of phylogenetic data for entelegyne spiders (Araneae: Araneomorphae: Entelegynae) with comments on their phylogeny. Proc Calif Acad Sci. 2005;56(Supplement II):1–324.
33. Kronestedt T, Marusik YM. Studies on species of holarctic *Pardosa* groups (Araneae, Lycosidae). VII. The *Pardosa tesquorum* group. Zootaxa. 2011;3131(1):1–34.
34. Kronestedt T, Marusik YM, Omelko MM. Studies on species of holarctic *Pardosa* groups (Araneae, Lycosidae). VIII. the palearctic species of the *Pardosa nigra* group. Zootaxa. 2014;3894(1):33–60.
35. Lopardo L, Hormiga G. On the Synaphrid Spider *Cepheia longiseta* (Simon 1881) (Araneae, Synaphridae). Am Museum Novit. 2007;20052(3575):18.
36. Lopardo L, Hormiga G. Out of the twilight zone: Phylogeny and evolutionary morphology of the orb-weaving spider family Mysmenidae, with a focus on spinneret spigot morphology in symphytognathoids (Araneae, Araneoidea). Zool J Linn Soc. 2015;173(3):527–786.
37. Marusik YM, Özkütük RS, Kunt KB. On the identity and distribution of the poorly known spider *Orthobula charitonovi* (Mikhailov, 1986) (Aranei: Corinnidae). Arthropoda Sel. 2013;22(2):157–62.
38. Schendel V, Junghanns A, Bilde T, Uhl G. Comparative female genital morphology in *Stegodyphus* spiders (Araneae: Eresidae). Zool Anz. 2018;1–10.
39. Álvarez-Padilla F, Hormiga G. A protocol for digesting internal soft tissues and mounting spiders for scanning electron microscopy. J Arachnol. 2008;35:538–42.
40. Kovblyuk MM, Kastrygina ZA, Marusik YM. New data on *Hahnia* C.L. koch, 1841 from Crimea (Aranei: Hahniidae). Turkish J Zool. 2017;41(2):311–7.
41. Burger M, Graber W, Michalik P, Kropf C. *Silhouettella loricatula* (Arachnida, Araneae, Oonopidae): a haplogyne spider with complex female genitalia. J Morphol. 2006;267(February):663–677.

42. Saaristo MI. Revision of the genus *Maro* O. P.-Cambridge (Araneae, Linyphiidae). Ann Zool Fennici. 1971;8:463–82.
43. Saaristo MI. Redelimitation of the genus *Oreonetides* Strand 1901 (Araneae Linyphiidae) based on an analysis of the genital organs. Ann Zool Fennici. 1972;9:69–74.
44. Saaristo M, Tanasevitch A V. Redelimitation of the subfamily Micronetinae Hull, 1920 and the genus *Lepthyphantes* Menge, 1866 with descriptions of some new genera (Aranei, Linyphiidae). Berichte des Naturwissenschaftlich-Medizinischen Vereins Innsbruck. 1996;83:163–86.
45. Uhl G, Gunnarsson B. Female genitalia in *Pityohyphantes phrygianus*, a spider with a skewed sex ratio. J Zool London. 2001;255:367–76.
46. Wang F, Ballesteros JA, Hormiga G, Chesters D, Zhan Y, Sun N, et al. Resolving the phylogeny of a speciose spider group, the family Linyphiidae (Araneae). Mol Phylogenet Evol. 2015;91:35–49.
47. Wang X-P. A Generic-Level Revision of the Spider Subfamily Coelotinae (Araneae, Amaurobiidae). Bull Am Museum Nat Hist. 2002;269(1):1–150.
48. Marusik YM, Omelko MM, Koponen S. First record of *Diphya wulingensis* Yu, Zhang et Omelko, 2014 (Aranei: Tetragnathidae) in Russia. Arthropoda Sel. 2017;26(2):139–44.
49. Eberhard WG, Huber BA. Courtship, copulation, and sperm transfer in *Leucauge mariana* (Aaraneae, Tetragnathidae) with implications for higher classification. J Arachnol. 1998;26(3):342–68.
50. Carico JE, Holt PC. A comparative study of the female copulatory apparatus of certain species in the spider genus *Dolomedes* (Pisauridae: Araneae). Terchnical Bull. 1964;172:1–27.
51 . Ramírez M. The spider subfamily Amaurobioidinae (Araneae, Anyphaenidae): a phylogenetic revision at the generic level. Bull Am Museum Nat Hist. 2003;277:1–262.

Supporting information

S1 Fig. Sections cross spermathecae of entelegyne spiders. A−B, *Neriene emphana* (Linyphiidae); C, *Parasteatoda tepidariorum* (Theridiidae); D−E, *Hahnia zhejiangensis* (Hahniidae); F, *Neoscona* sp. (Araneidae). CD, copulatory duct; S_1, primary spermatheca; S_2, secondary spermatheca; SD, spermathecal duct. Scale bars: 0.1 mm.

S1 Table. Collecting data of materials examined in this study.

S2 Table. Epigynal tracts and mating plugs in entelegyne spiders.

Figure

Click here to access/download;Figure;Fig 1. Epigynum and mating plugs of Diphya wulingensis.tif

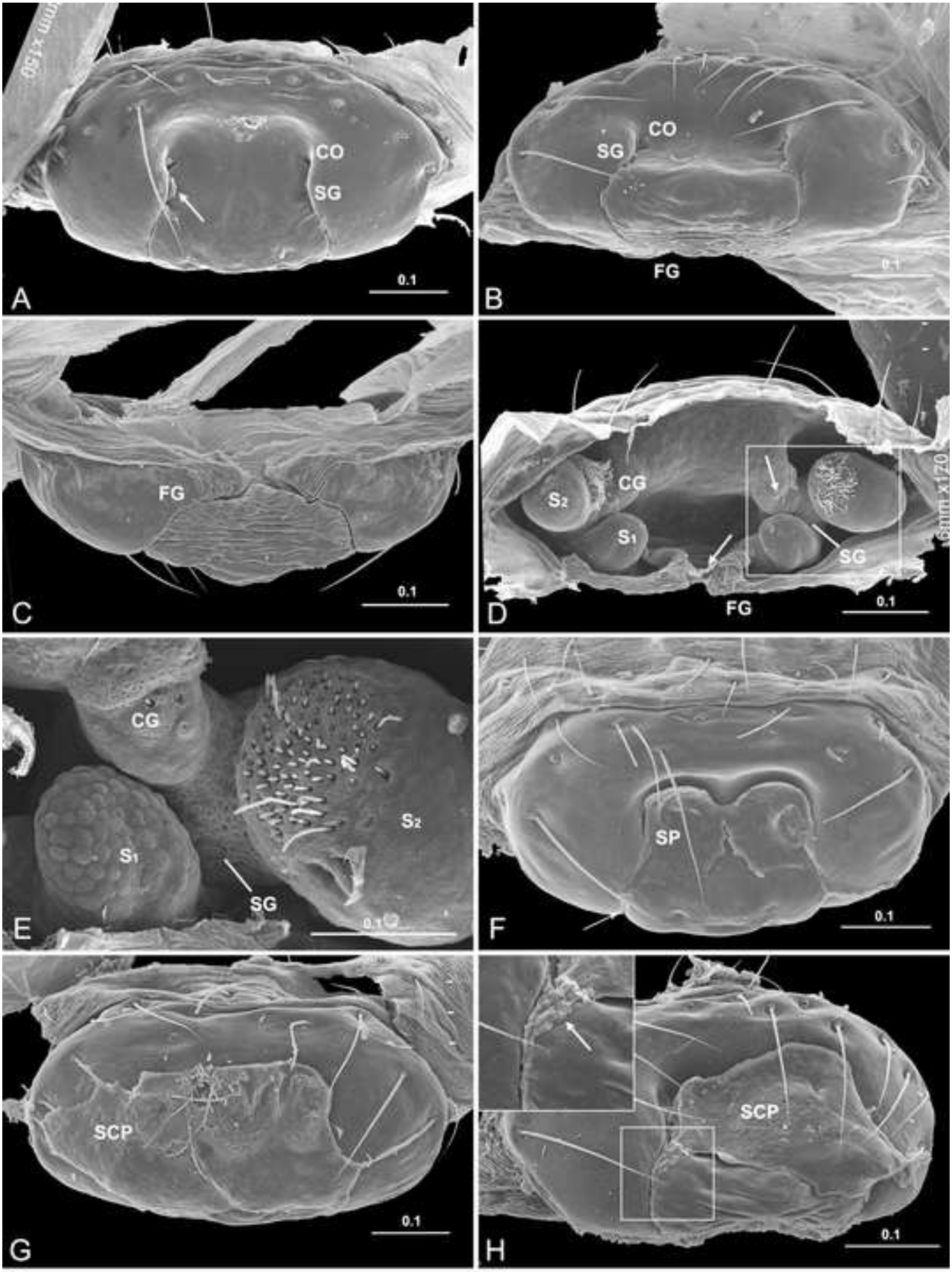

Click here to access/download;Figure;Fig 2. Cross sections of epigynum of Diphya wulingensis.tif

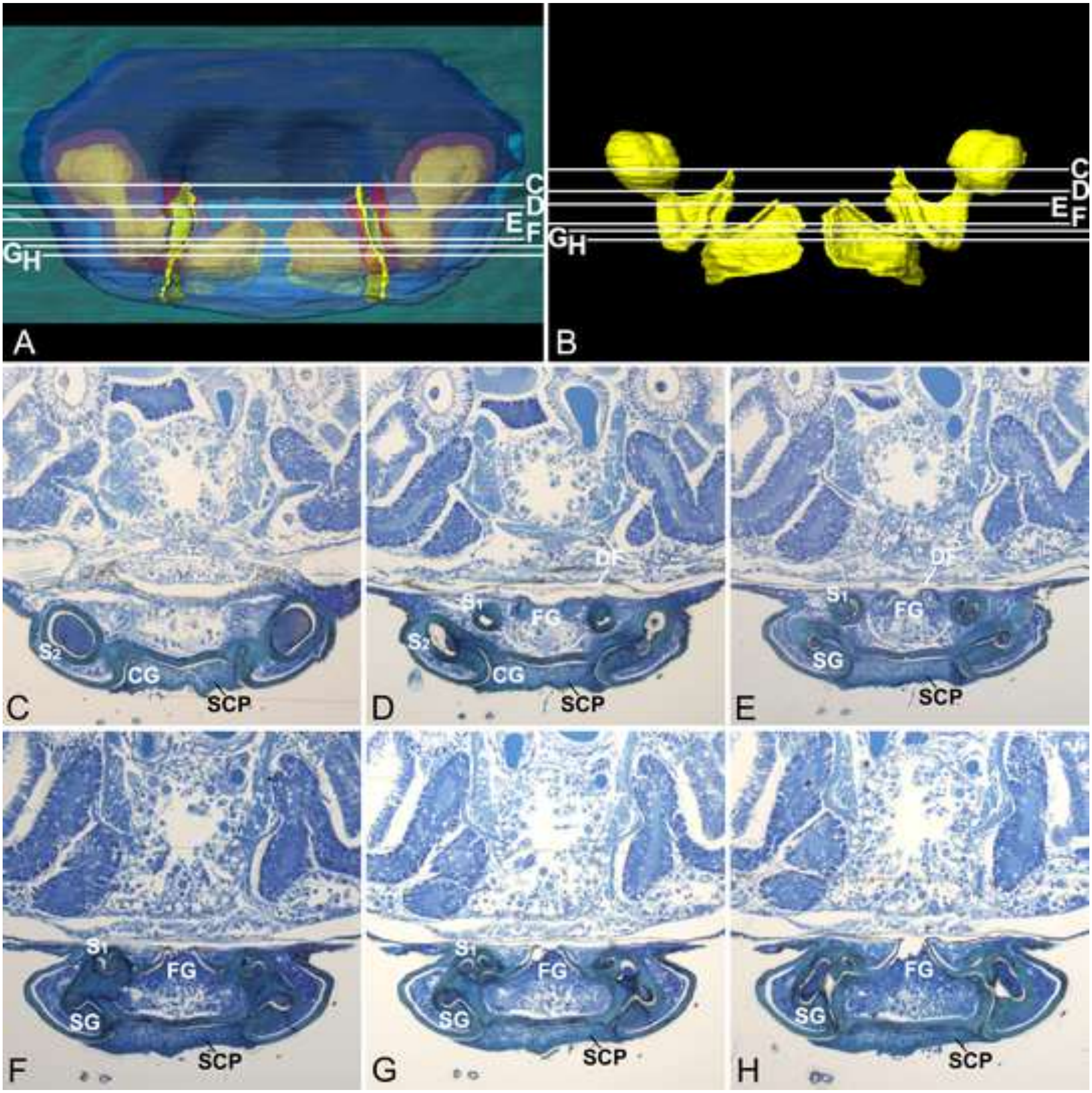

Figure

Click here to access/download;Figure;Fig 3. 3D-reconstructed epigynum of Diphya wulingensis.tif

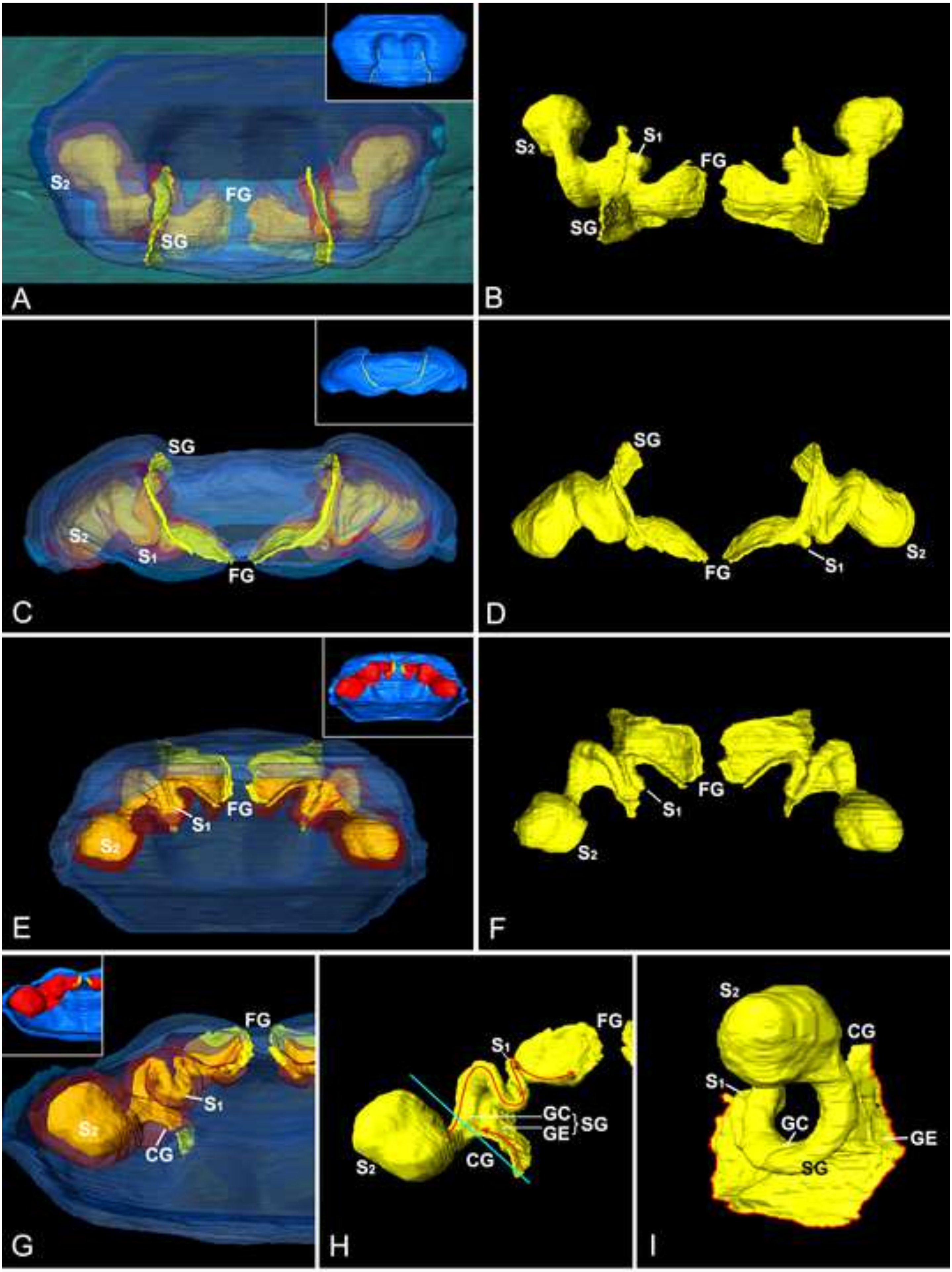

Click here to access/download;Figure;Fig 4. Schematic structure of epigynal tract.tif

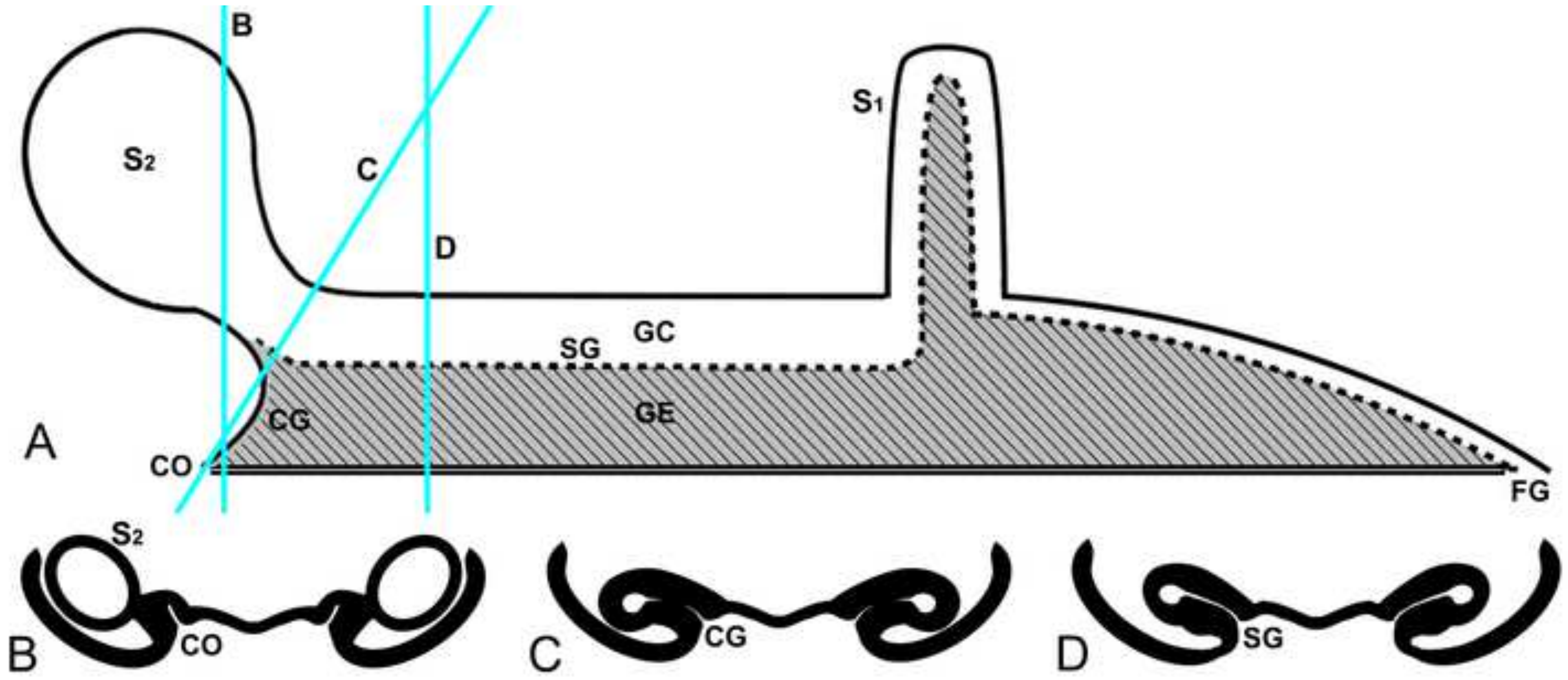

Click here to access/download;Figure;Fig 5. Types of entelegyne spermathecae.tif

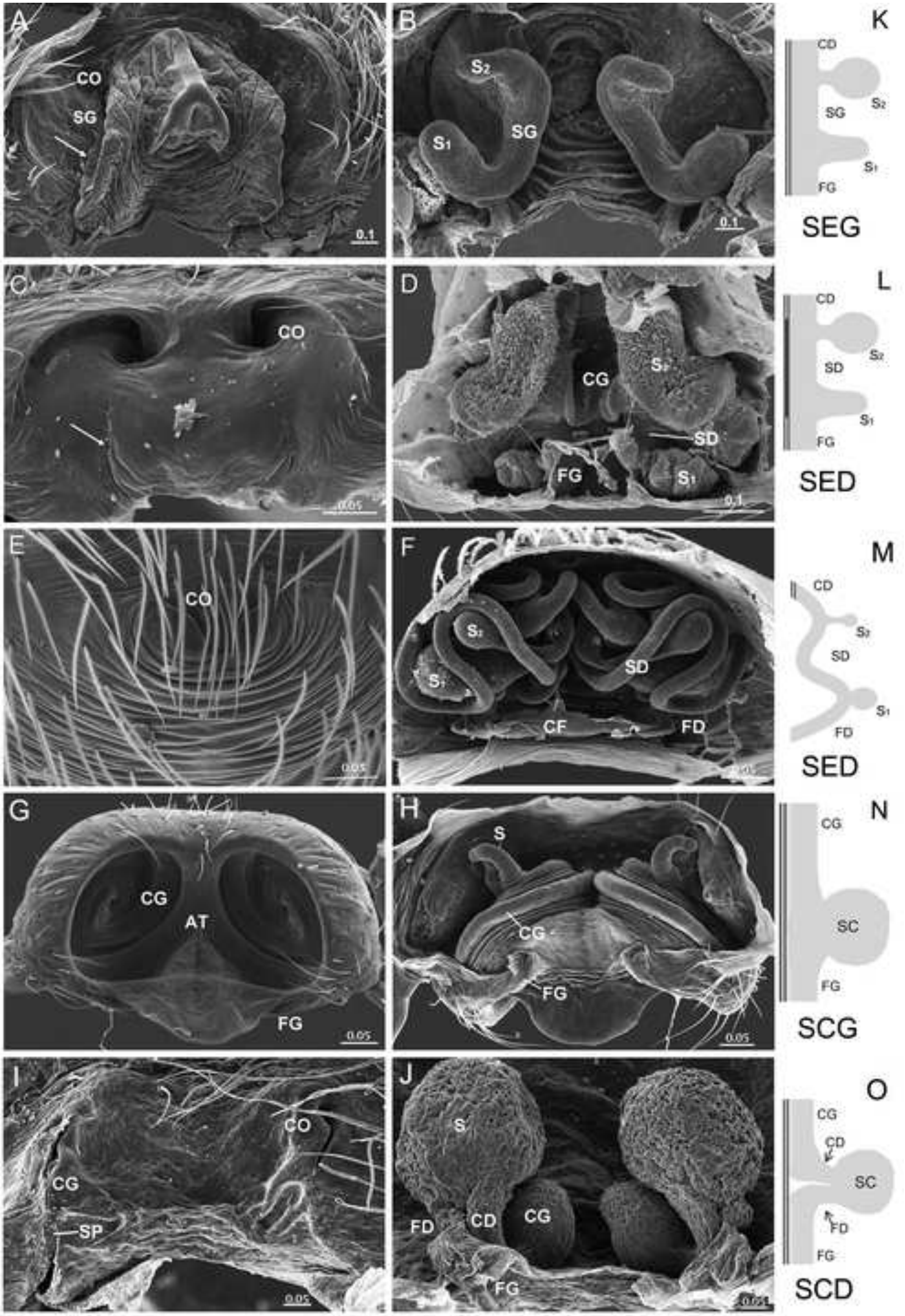

Figure

Click here to access/download;Figure;Fig 6. Variation in elongated spermathecae.tif

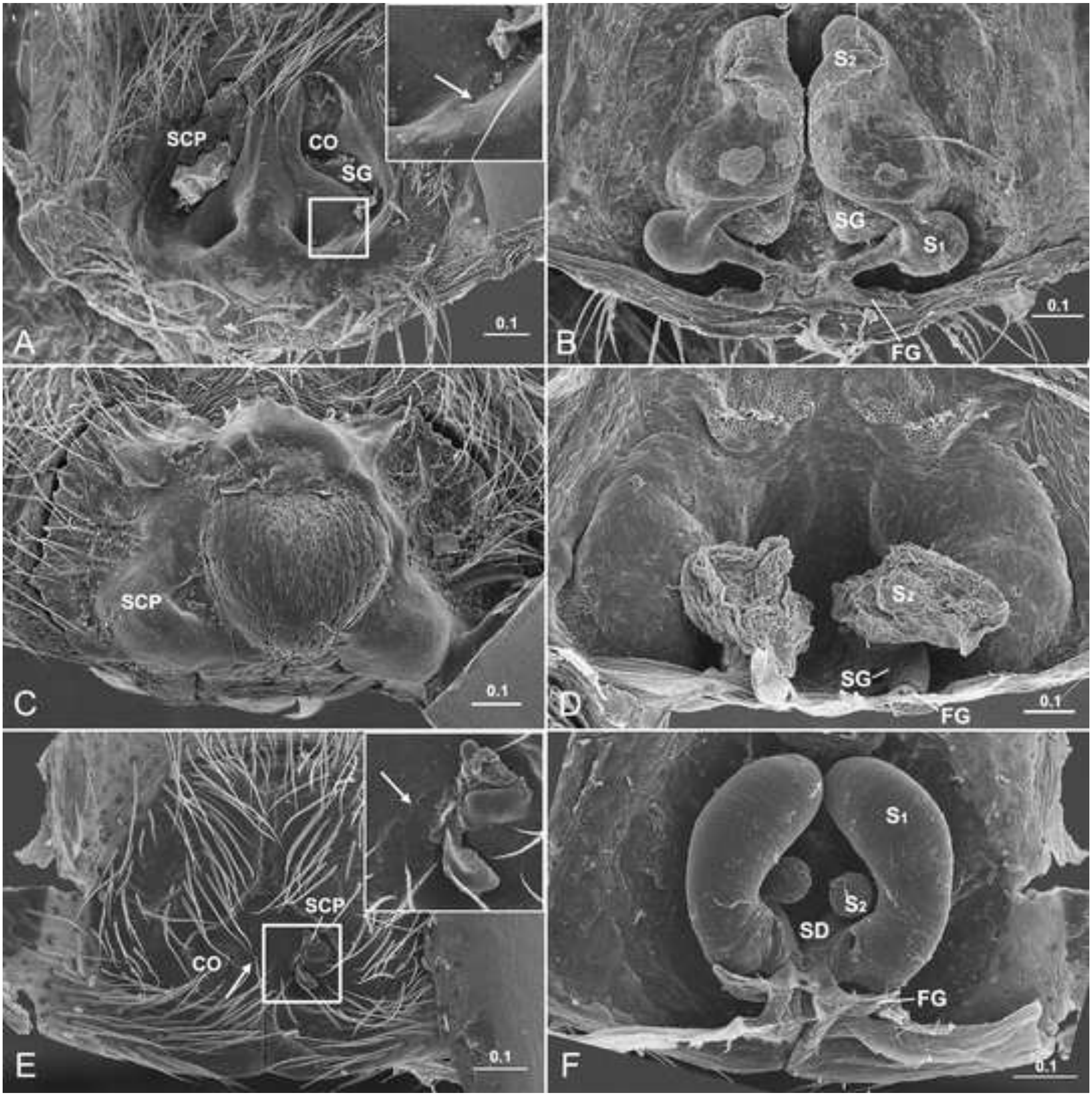

Figure

Click here to access/download;Figure;Fig 7. Mating plugs in entelegyne spiders.tif

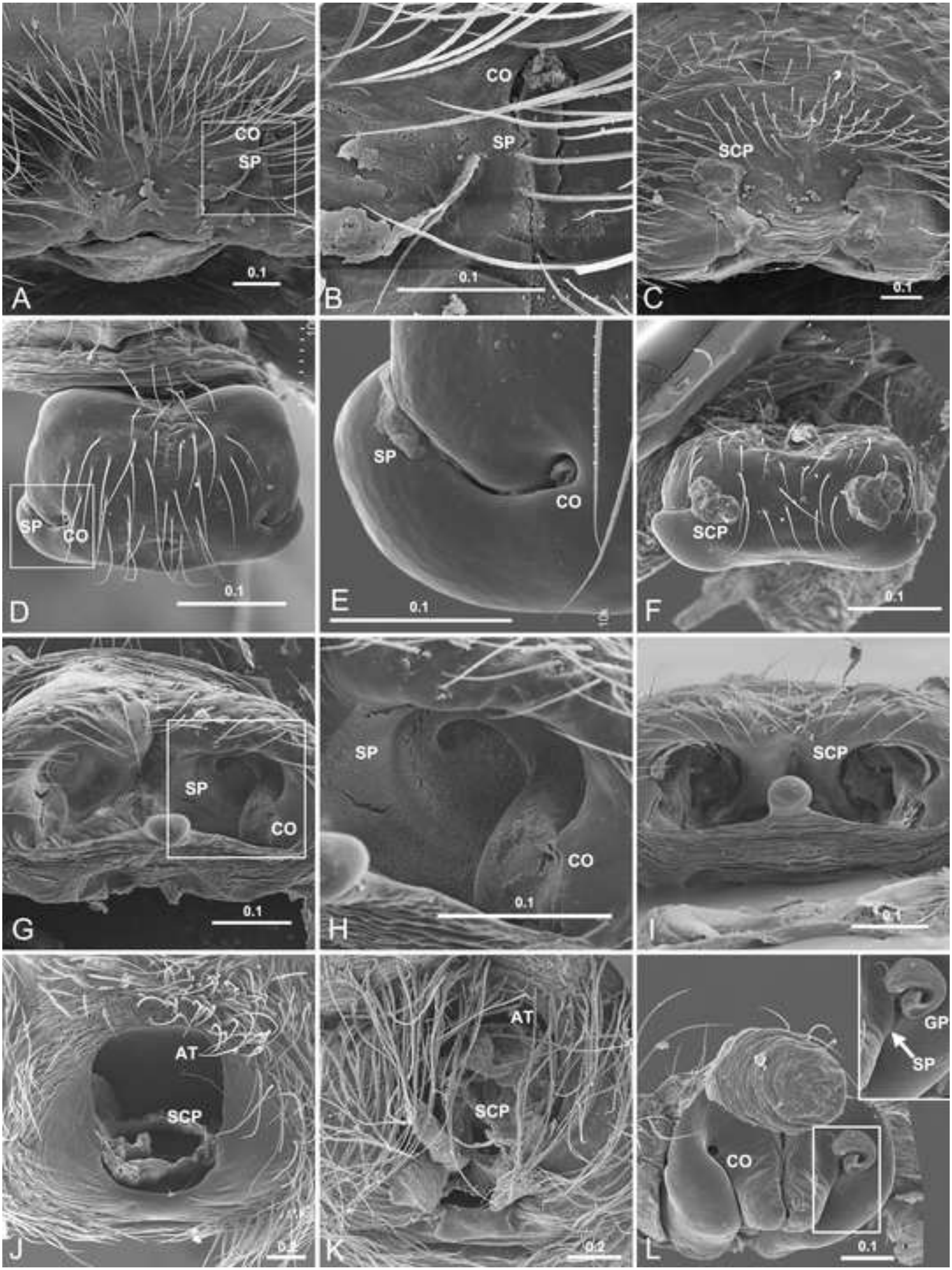

Click here to access/download
Supporting Information
S2 Table. Epiginal tracts and mating plugs in spiders.xlsx

Click here to access/download
Supporting Information
S1 Fig. Sections cross spermathecae of entelegyne spiders.tif

Click here to access/download
Supporting Information
S1 Table. Collecting data.docx
