# Supplementary material for: A spider mating plug functions to protect sperm
Source: PLoS One. 2024 Mar 29;19(3):e0301290. doi: 10.1371/journal.pone.0301290 (PMC10980215; doi:10.1371/journal.pone.0301290)
Supplement: Attachment — Submitted filename: 02.Response to Reviewers-20240103.docx [file pone.0301290.s005.docx]

PONE-D-22-34374

A spider mating plug functions to protect sperm

PLOS ONE

5. Review Comments to the Author

Please use the space provided to explain your answers to the questions above. You may also include additional comments for the author, including concerns about dual publication, research ethics, or publication ethics. (Please upload your review as an attachment if it exceeds 20,000 characters)

**Reviewer #1: This manuscript includes a large amount and detailed work analyzing morphological structures on female genitalia in spiders. On the one hand, authors study in detail the morphology of epigynal tracts in the spider Diphya wulingensis. They found two types of mating plug: sperm plug and secretion plug, and suggested these plugs serve for different functions. The sperm plugs function as temporary protection mechanism to prevent sperm from leaking and desiccating, whereas the secretion plug act for permanent protection mechanism but also for preventing additional matings. On the other hand, they perform a comparative study on spermathecal morphology using several spiders to test whether there are multiple resolutions to sperm protection.**

**I think the manuscript is very valuable and has an interesting and broad evolutionary approach.**

Thank you for your affirmation of our work.

**However, my first and main concern is that I was not really convinced on the idea of the plug as sperm protection. Authors do not present direct evidence on how sperm is protected by the plugs.**

The main reasons for the sperm leakage are that there is no closing mechanism inside the spermathecal entrance and exit, as well as the slit between them. There are two ways to solve the problem in *Diphya wulingensis*: 1) SCP covers the entire epigynal plate, simultaneously blocking the copulatory openings and the spermathecal groove slits (Fig. 1G); 2) The epigynal tract wall modified into a 'tadpole shaped ' in cross section, forming a ribbon like groove edge between the groove slit and groove chamber from beginning to end, including the part of the spermathecae (Figs 2, 3). The fragments of scab formed by the spilled sperm are embedded in the groove slits (Fig. 1A, B). We infer, the two tightly attached layers of the groove edge capture the spilled sperm liquid that will seal the groove slit under the action of surface tension. Therefore, the so-called sperm plug (SP) needs the joint action of spilled sperm and the modified spermathecal groove walls. The scabs on the epigynal plate (Fig. 1F) and the sperm particles under SCP (Fig.1H) are evidence of sperm leakage, indicating that there was indeed a problem of sperm leakage before the formation of SCP. Based on the characteristics of mating plugs and females multiple mating, we proposed that SP is a temporary sperm protection mechanism that acts during the intermating period, while SCP is permanent and acts during the post mating period.

**I understand morphologies can give us indirect evidence to postulate functions but the actual role of the plugs need to be tested using physiological and behavioral approaches.**

Our comparative approach outlines the directions for future experimental tests. To demonstrate that a mating plug serves as a mating strategy, the most common mating experiments usually measure whether the plug presence can decrease the ratio of female remating. However, for *D. wulingensis*, there are two problems:

1) The secretion plugs in *D. wulingensis* are stable enough to form an obstacle for female remating. Nevertheless, mating experiments fail to resolve the questions: Why the plug material needs to cover the entire epigynal plate, rather than only block the copulatory openings?
2) What is the driving force that promote the spermathecal wall modified into such a ' tadpole shaped ' in cross section?

Since there are two hypotheses about the function of mating plugs, and in many case studies, both functions are possibal, even if the results of mating experiments show that the existences of mating plugs can significantly decrease the rate of female remating, the possibility of the plug formation being used for sperm protection has not been considered or ruled out (Line 107). Although we have not directly demonstrated that the existence of mating plugs can decrease the rate of female remating, according to our predictions based on two hypotheses, our results are accord with the prediction of the sperm protection hypothesis, thus proving that the formations of SCP and SP are intended as sperm protection mechanisms.

**As far as I followed the text, there is no references for the main problem exposed here in relation to the potential leakage and desiccation of sperm after copulation. I think this information and examples on how sperm is “unprotected” needs to be at the first line of arguments.**

Indeed, this is an obvious but often ignored issue, rarely addressed in previous studies. In **Introduction**, we pointed out that the spermatheca with separated entrance and exit and spermathecal wall highly sclerotized are common characters for the entelegyne spermathecae, no closing mechanism have ever been reported, thus all entelegyne spiders have to deal with the risk of sperm leakage, desiccation and backflow caused by the structural characteristics of the entelegyne spermathecae (Line 68). In *D. wulingensis*, sperm leakage really occurred in those individuals without secretion plugs formed (Fig. 1F, H), also in many other spiders (Table S2). As a successful terrestrial animal group, the sperm of spiders must be well protected, but we are not sure what this protective mechanism is. Since Huber (1995) proposed that mating plug may be a potential sperm protection mechanism, few empirical tests have emerged (but see Tian et al. 2023).

For those spiders have no mating plugs found in this study, it does not mean that their sperm is “unprotected”. Because mating plugs were examined externally from the epigynal plate, most samples were not examined whether their spermathecal entrances have plugged or not. Similar situations are common in previous studies. We have argued this in “Introduction” (Line 105).

**In relation to the focal species there is lack of information on whether female mate multiple and how the plug affects female willingness to remate, as well as males pedipalp insertions and mating duration. Some kind of theoretical construction around this issue needs to be done in the introduction to better understand the connection with the morphological findings on the study species.**

SCP in *D. wulingensis* is very sturdy and durable, we do not query whether it is sufficient to constitute a mating obstacle that needs to be tested through mating experiments. While what we are interested in are its special spermathecal structure and the special features of mating plugs, these help us verify whether the formation of SCP is to impede female’s remating, or to protect sperm. We conducted morphological study on its epigynum to analyze why the plug material had to cover the entire epigynal plate, what driving force that promote the wall of spermathecal groove modified in such a way, and what modifications in spermathecal structure resolved the contradiction between preventing sperm leakage and not hindering female’s remating.

**Finally, I need to admit that it was absolutely tedious to read the results and follow the main idea tested, so I suggest to revise the results section in order to improve clarity for readers.**

The morphological description is indeed boring, though we tried our best to improve the readability of the manuscript, and the figure plates can of course be helpful.

**For the comparative study across different species, I was expecting to see a phylogeny with the different mating plugs, which it would help to see the topic in broader way.**

A phylogenetic approach is slightly out of the scope here, but our information strongly suggests high levels of homoplasy in variation of epigynal structures in spiders. Clearly, mating plugs originated in spiders multiple times.

**Main comments

Introduction
I think this section needs more work. I suggest to re-write it, giving evidence on sperm viability, mobility and endurance along time in spiders. This can help to build arguments on the problem of dissection.**

Thanks for your constructive suggestion. We have rewritten in Introduction as: “Newly published study on the spider *Holocnemus pluchei* found (Cargnelutti et al. 2021): 1) the viability of sperm stored in female’s genital tracts decreased than that in male; 2) However, such a decrease has little relation to the length of time for store; 3) the sperm from the males having performed a longer post-insemination behavior had higher sperm viability inside the female. These imply that the female storage might negatively affect the sperm viability (Herberstein et al. 2002; Cargnelutti et al. 2021) although the reasons that lead to sperm viability decreased are not clear. There are multiple possibilities, such as the quality of the sperm itself (Rodriguez de Souza et al., 2020), the issue of sperm aging (Reinhardt Review, 2007), and the trade-off between the female immune system and maintaining stored sperm motility (Rolff & Siva Joseph, 2003), as well as sperm competition or cryptic female choice that take place in the female genital tracts (Engqvist, 2012; Cargnelutti et al., 2020; Birkhead et al., 1993; Pitnick et al., 2009; Fitzpatrick & Lüpold, 2014). Nevertheless, it cannot be ignored that the inherent defects in the structure of the entelegyne spermathecae pose the risk of leakage and desiccation to the stored sperm. The decrease in sperm viability within the female tracts did not worsen over time, indicating effective risk control. Among the possible post-insemination behavior, it has been proposed to act as post-copulatory mate guarding (Kuntner et al. 2009c; Calbacho-Rosa et al., 2010; Lee et al. 2012) or copulatory courtship (Huber & Eberhard,1997; Schäfer & Uhl, 2002; Peretti & Eberhart, 2010; Calbacho-Rosa & Peretti, 2015), dedicating to generate mating plugs (Knoflach 2004; Tian et al. 2023) might be the most likely help to maintain sperm viability. Huber (1995) proposed that mating plugs may be a potential sperm protection mechanism, but there has been a lack of empirical evidence.

**Further, information on sperm leaking is needed.
Also, this section needs to have a paragraph on the sexual behavior of the focal species.**

As mentioned above, the fragments of scab formed by the spilled sperm are embedded in the groove slits (Fig. 1A, B), the scabs on the epigynal plate (Fig. 1F) and the sperm particles under SCP (Fig. 1H) are evidence of sperm leakage. The secretion plugs lodged on the spilled sperm indicate that sperm leakage really took place before the formation of secretion plugs.

These are presented in the first paragraph of page 15 (Line 259) and Figure 1.

**In relation to the main hypothesis, what about if the very large and extensive plugs (here called SCP) found in some species are subject of intense sexual selection? I can imagine that males can leave a sperm plug (SP) under low sperm competition and additionally a SCP under high sperm competition. What happens with the spider species that don’t have mating plugs? Are these species having less sperm for fertilization? In my opinion, these possibilities need to be discussed at the same level than the sperm protection hypothesis.**

This is a good point. We indeed study mating plugs in some other spiders. In *Trichonephila clavata*, an extremely sexually size dimorphic spider charactered by biased sex ratio in the population, both sexes are subjected to intense sexual selection. We found four types of mating plugs in this species, two types of secretion plugs, two types of genital plugs, and the spider might use different type of mating plugs to solve different problems. We also explore *Neriene emphana*, a linyphiid under low sperm competition (Tian et al. 2023). We found the occurrence ratio of SCP is 100% in mated females, and its target is to block spermathecal entrance. A sperm protection mechanism is needed by all female individuals, and hence one may find the formation of SCP is an obligate part of copulation in *N. emphana.* In all these three spiders, it is mating plug serving as sperm protection mechanism, but working in different ways.

The spiders in this study were randomly sampled, unexpectedly found high occurrence of mating plugs. Individuals of *D. wulingensis* are 100% plugged, either SP or SCP, or both. This is attributed to: 1) densely sampled; 2) the samples were examined by SEM; 3) most materials of *D. wulingensis* only treated by water cleaning to avoid the plugs damaged during material preparation. For those spiders who have not been checked out mating plugs, most of them were only examined one sample, or virgins, and the epigyna were treated by enzymolysis and supersonic cleaner that may damage the plugs. The most important is that if mating plugs function as a sperm protection mechanism, their plugging targets should be spermathecal entrances. In most cases, mating plugs are detected externally from copulatory openings, it is not known whether the spermathecal entrances are blocked simultaneously or not (Line 106). Therefore, we may infer that in most of spider species mating plugs may not be really absent, but not found. Our results indicate that mating plugs serving as a sperm protection mechanism might be common in spider.

The sperm protection mechanisms of *D. wulingensis* can only represent that of those species with the SEG type spermathecae, one of the four types of the entelegyne spermathecae. It needs to deal with the sperm protection problems of both spermathecal entrance and groove slits, while for the other three types of spermathecae, changes in the structure of the spermathecal grooves, such as shortened groove length, or groove slit becoming closed, or inward displacement of the spermathecae, etc, resolved the sperm leakage and desiccation from the spermathecal groove slits. To prevent sperm leakage and desiccation from the spermathecal entrances, we did not find any resolutions of sperm protection, other than mating plugs. Nevertheless, mating plugs in spider not only have different appearances, different compositions. different formations, different origins, but also function in different ways. Therefore, we cannot arrive at an overall conclusion now.

**Results. This section can be improve with subheadings.**

Agreed, added.

**Discussion.
This section can be improved following the main suggestion on the introduction.**

**Minor comments

L30-49. The abstract lacks explicit information on the comparative study**

Added.

**L88-90. These are very odd figure numbers and I couldn’t find the figures. It doesn’t need to be here if the information is from the published papers.**

The number in [ ] are the number of references in **References**. The figure number corrected.

**L94-98. Develop the main findings from Huber’s paper.**

Huber (1995) only suggested considering another possibility: **“**Such plugs have been found in a great variety of spiders (Austad, 1984) and have often been interpreted as devices of paternity assurance. However, detailed investigations would be needed to show whether such plugs really can influence further insemination attempts (Jackson, 1980; Masumoto, 1993) or whether alternative hypotheses should be considered (e.g. simply superfluous sperm; protection against desiccation of sperm in the spermatheca; protection against flowout of sperm).”

**L152. Here you stated 19 females but in L280 you sated 17 females. Please revise it.**

A total of nineteen epigyna were examined in this study, including seventeen by SEM and two for histological study by semi thin HSS (Line 153). The seventeen epigyna in Line 280 refer to those examined by SEM.

**L192. I suggested the subheading (or similar): Female genital anatomy and sperm storage**

Thanks, added.

**L194-255. This first section is descriptive, which is fine but very tedious. I suggest incorporate information on the function of the structures to help readers to follow it.**

Yes, descriptions of complex genital structures are very tedious in general, but are necessary for readers to understand their functions. Based on these, we discussed why in *D.* *wulingensis* the secretion plugs lodge at copulatory openings or covering the whole epigynal plate have different biological meanings, and both SCP and SP are in line with the prediction of the sperm protection hypothesis (Line 358); how the modified wall of spermathecal groove together with the spilled sperm form a temporary sperm protection mechanism (Line 304) that would not prevent sperm to be released from S_2_ and would not being an obstacle for a subsequent male, therefore not prevent female polyandry (Line 409).

**L259. I suggested the subheading (or similar): Sperm and secretion plugs**

Added.

**L280-287. This information needs to be at the beginning of the section.**

This information is about the examining results of mating plugs in *D. wulingensis*. It was placed here due to its description requiring the foreshadowing of the division of mating plug types based on the characteristics of plug materials, and its placements (site and deposited features) based on the structural characteristics of epigynal tracts.

**L289. Table 1. What is the difference between complete and intact?**

This is presented in Line 272-276.

**L291. I suggested the subheading (or similar): Copulatory plugs along the phylogeny**

We added two subheadings of ‘Four types of entelegyne spermathecae’ and ‘Mating plugs in entelegyne spiders.’

**L291. Here you stated 394 epygina from 330 species, but in L317 you stated 137 out of 411 mating plugs. How did you reach these numbers? I think it is important to put all the information together to understand the sample sizes.**

‘Besides *D. wulingensis*, additional 394 epigyna from 330 species, 187 genera, 36 families were also examined here’ (Line 291). The 411 materials examined here including 17 epigyna of *D. wulingensis* and 394 epigyna of other spiders. All this information is presented in S2 Table.

**L291-312. Please indicate the name of the epigyna type in bold**

Added.

**L318. This is the first time you mentioned genital plug in the text. What is the difference between mating and genital plug? Before you defined sperm plug and secretion plugs, I am confused.**

We introduced ‘Mating plugs refer to either genital pieces (hereafter genital plug) or hardened secretions (hereafter secretion plug) lodged in copulatory openings during or after copulation’(Line 79). In *D. wulingensis* we did not find genital plugs.

**L291-334. It is important to include the phylogeny and map the type of mating plugs.**

Given the great diversity of epigynal characters, multiple originations and multiple functions of mating plugs, as well as multiple interaction models between epigynal tracts and mating plugs, we have not obtained enough data to explore this issue based on a phylogenetic frame work.

**L338-340. Is there any evidence showing that these problems exist?**

As mentioned above, when spiders mate normally, sperm should be protected, but we do not know what this protective mechanism is. So far, there has been no clear evidence of sperm leakage reported in published literature, but in our SEM images, sperm leakage is not rare. There are several situations: 1) such as *D. Wulingensis* (Fig. 1A, B, F, G), the overflowing sperm combines with the deformed spermathecal wall to seal the slit like opening; 2) Spilled sperm granules are embedded in the groove slits, sealing the slit-like openings. Such a situation is very common in linyphiid spiders with groove-shaped epigynal tracts and SCG type of spermathecae (CG-SCG-FG, Tu unpublished data); 3) The mating process is artificially interrupted, and sperm leakage would occur if SCP has not been formed (Tian et al. 2023); 4) Many scabs and clusters of sperm particles scattered on the epigynal plate due to the copulatory openings and grooves having been blocked by SCP, and the subsequent male fail to inject successfully. This situation is seen in the extremely sexually size dimorphic spiders with high sexual selection pressure, such as *Trichonephila clavata* (Jiang et al. in prepare).

**L341. Please add a reference on the amount and diversity of spiders**

Added.

**L387. I don’t think you can give an intentionality**

Comparing the states of SP and SCP substances, you’ll find the formations of the two plugs are different. The spermatheca has a slit-like opening between the entrance and exit, thus under the pressure of injection, sperm leakage is a natural phenomenon. The spilled sperm captured by the two layers of the spermathecal groove edge, seal the slit under the surface tension. But the generation of SCP is intentional: 1) The dense secretion that forms SCP is intentionally transported to the epigynal plate, rather than naturally spilled or occurring unexpectedly; 2) The complete SCP surface is almost flat (Fig 1G), covering the entire epigynal plate, not as a naturally dry out state of secretions with a uneven suface (Fig 1H), this indicates that it was intentionally done.

**L388. Did you perform matings? how do you know females were mated?**

We did not perform mating trials in *D. wulingensis*. We determined that these female spiders have already mated based on the presence of mating plugs, either SP or SCP, on their epigynal plate.

**L409-410. How do you know?**

Please see the paragraph explaining why.

**L426-433. This information needs to be placed before**

The information here is not simply a description of the interaction between male palpal embolus and female spermathecal entrance during copulation, but rather a demonstration of the interaction mode between them. Combined with the foreshadowed characteristics of SP and SCP, and the spermathecal features, we try to explain, 1) for the S_2_ with a single opening, why the SP used for sperm protection during the intermating period would not being an obstacle for subsequent mating, 2) why the SP and SCP blocking the spermathecal entrance would not affect the sperm release from S_2_ into the spermathecal groove,

**L464-465. I don’t understand this sentence.**

We suggest that all entelegyne spiders require a means of sperm protection, while a mating strategy only has significance for polyandrous spiders. Because not all spiders engage in polyandrous mating, mating plugs commonly present in spiders may imply that mating plugs may have common use as a sperm protection mechanism, in addition to be a mating obstacle.

**Reviewer #2:**

**The manuscript documentation is of excellent quality, in particular the histology cuts and 3D reconstructions. I consider that the manuscript is publishable. The following ideas are optional and I think will make the paper even better.**

Thanks for your affirmation of our work and your comments.

1) **The authors talk about testing the hypothesis whether SP and SCP could have evolve for sperm protection in addition to prevent sperm competition. The anatomical description of the different genitals systems is very thorough and useful, but it only focuses in spiders that usually present plugs. Do you consider a descriptive comparison with epigyna of species that do not present them is relevant?**

Our comparative morphological study mainly focuses on the spermathecal features:

a) the spermathecae have double opening and the spermathecal groove walls highly sclerotized are two common features of all entelegyne spiders, the issue of sperm protection is a common problem for all entelegyne spiders.
b) However, the results of comparative study of spider epigyna suggest there are multiple solutions to the problem of sperm protection: Structural changes in the spermathecae, such as deformation of the spermathecal wall, fusing and shortening of the spermathecal groove slits, and inward placement of the spermathecae, can help solve the problem of sperm leakage from the spermathecal groove slits. Only for those spiders of SEG type spermathecae, the sperm protection mechanism has to simultaneously solve the problem of leakage from the spermathecal entrance and the spermathecal groove slits. Although the structural changes of the other three types of spermathecae are different, their common result is to minimize the impact of the spermathecal groove slits, only retain the entrance and exit. This also suggests the necessity of a protective mechanism for spermathecal groove slits.

In most cases, mating plugs were unexpectedly detected from the copulatory openings. We even not sure those spiders not found plugs in this study are really have no mating plugs. Our results of comparative study on epigynal tracts show that mating plugs serving as a sperm protection mechanism might common in spiders. Of cause, this needs to be tested in future study.

2) **In the S2 Table you include species that present plugs and species that do not in addition to the types of genital systems described. Do you consider useful to apply some statistical analysis with these data? I think that will give more support to the testing part of the paper.**

The epigyna examined in this study were randomly sampled for exploring spermathecal morphology, unexpectedly found high occurrence of mating plugs, especially secretion plugs. Increasing individual samples in some spiders show the plug ratio even higher. Therefore, for most species with one sample, no plug detected does not imply definite absence in this species. Furthermore，that more than one type of mating plug was found in some spiders，implies they may function in different ways. To deeply explore the function and other biological questions of mating plugs, applying some statistical analysis is necessary but within a different focus. On the other hand, statistical analysis requires developing different sampling strategy for different problems. We applied statistical analysis in other studies, e.g. Tian et al. (2023).

3) **Do you consider relevant to mention volumes of the different spermathecal cavities, ducts or groves? This of course depend how difficult is to get these data. Is this data possible to obtain from the 3D reconstructions?**

Theoretically, it is feasible because the walls of the spermathecae are highly sclerotized. Treatments in specimen preparation following Álvarez-Padilla and Hormiga (2008) will have little effects on the spermathecal shape. While not done in this paper, it is possible to calculate the volume of the chambers through image measurement. The spermathecal structures in some spider groups vary a lot in the number of spermathecal chambers, the shape of spermathecal cavity, and the spermathecal tracts in duct or groove state. These might affect the pattern of sperm flow in the spermathecae, and the problem of sperm protection might have different resolutions. This article is a case study based on the special features of the spermathecae of *D. wulingensis.* Further studies for other spider species in future, the impact of these factors can be considered.

**Comments in the attached file PONE-D-22-34374_reviewer_FAP**

**Line 67. Consider mentioning the case of some pholcids that present haplogine genitalia and marting plugs.**

Added.

**Line 91. Hello Matjaz, do you remember the SEM image of a Nephila mating pulg perforated with several emboli, if you find it it will look very nice here.**

Added.

**Line 439. Only with ontogenetic evidence? Please consider revising a relevant phylogeny otherwise this conclusion could be dangerous.**

Thank you for the reminder, we have rewritten it. In addition to ontogenetic evidence, there are also morphological features as evidence to support our conclution. The discussion based on a relevant phylogeny needs to be addressed in a separate article.

**Line 474. What happens in entelegyne taxa that do not present SP or SCP. It would be very informative to include the anatomy of such species in contrast to the ones described here.**

Although only 137 out of 411 samples detected mating plugs here, it far exceeds previous expectations based on mating plugs being a mating strategy hypothesis. Because it is a random sampling for morphological research, most species only examined one specimen. For those species that did not detect mating plugs, the following reasons are possible: 1) Virgin females who have not yet mated or have not completed mating, and have not yet formed mating plugs; 2) SP was damaged during specimen preparation; 3) The mating plugs are lodged inside the epigynal tracts, fail to be shown in the SEM images; 4) There may be other solutions. Therefore, for those species not found mating plugs do not mean absent. We cannot make sure any spiders that do not have mating plugs.

**Could it be possible to use the data in the S2 table to cover these comparisons? Also could it be possible to use the data in this table to make a chi square or other hypothesis test that relates internal genital architecture with SP or SCP?**

As we argued above, taxa in Table S2 were randomly sampled for morphological research, not suitable for statistical analysis. However, it includes many detail information of epigynal tract characters that may be used for other hypothesis test that relates internal genital architecture with SP or SCP.
