# Supplementary material for: A spider mating plug functions to protect sperm
Source: PLoS One. 2024 Mar 29;19(3):e0301290. doi: 10.1371/journal.pone.0301290 (PMC10980215; doi:10.1371/journal.pone.0301290)
Supplement: Attachment — Submitted filename: Comments for authors.docx [file pone.0301290.s006.docx]

**Comments for authors**

In the present manuscript, the authors test the hypothesis that mating plugs, in addition to being adaptations for mating strategies, may serve as a sperm protection mechanism. The manuscript is really well written and of quality. It is well organized and the data set supports, in part, the discussions and conclusions drawn by the authors. In my opinion, the manuscript deserves to be published after the aforementioned corrections and suggestions.

I see three main problems in the manuscript: The first is that although the writing is really well done and the topic is interesting, I think the authors should put a little more information about mating plugs in other taxonomic groups. Because it is a widely distributed journal, readers in areas other than arachnology may not see interest in the manuscript. I suggest then that the first paragraph of the introduction not go directly to spiders and rather be a very brief summary of mating plug studies in other taxonomic groups.

The second problem has to do with the excess of details in the results and the discussion. I understand the need to detail all the structures of the epigynium because the manuscript is through a morphological approach, but the idea is really lost a bit after two full pages of reading the very detailed description of the structures. I suggest that both the results and the discussion be shortened for the reader's convenience.

Finally, although the morphological approach is really good and the detail and comparison between species is really interesting, we know that they are still indirect evidence of the functions of the structures. I really don't know if the authors performed subsequent experiments with the individuals under laboratory conditions to corroborate the conclusions drawn in the manuscript.

**Abstract**

I understand that a large part of the study was focused on the species *D. wulingensis*, but I still feel that part of the main results is missing from the comparison that was made with the other species.

**Minor fixes**

- Page 3, line32: I think the authors should provide a little more information about mating plugs both in invertebrates (which is where it is best known) and in vertebrates. Because it is a widely distributed journal, readers from areas other than arachnology may not see interest in the manuscript. I suggest then that the first paragraph of the introduction not go directly to spiders and rather be a very brief summary of mating plug studies in other taxonomic groups.
- Page 3, lines 60 and 62, could you please put some references?
- Page 5, line 103: missing parentheses to close the sentence.

**MATERIALS AND METHODS**

**Study species**

I feel that some biological data is needed on the study species, *D. wulingensis*, I suggest placing in materials and methods a "study species" section on what is known about the species.
