# Supplementary material for: A spider mating plug functions to protect sperm
Source: PLoS One. 2024 Mar 29;19(3):e0301290. doi: 10.1371/journal.pone.0301290 (PMC10980215; doi:10.1371/journal.pone.0301290)
Supplement: Attachment — Submitted filename: Response to reviewer comments.docx [file pone.0301290.s007.docx]

PONE-D-22-34374R1

A spider mating plug functions to protect sperm

PLOS ONE

Reviewer #3: Comments for authors
In the present manuscript, the authors test the hypothesis that mating plugs, in addition to being adaptations for mating strategies, may serve as a sperm protection mechanism. The manuscript is really well written and of quality. It is well organized and the data set supports, in part, the discussions and conclusions drawn by the authors. In my opinion, the manuscript deserves to be published after the aforementioned corrections and suggestions.

1. **I see three main problems in the manuscript: The first is that although the writing is really well done and the topic is interesting, I think the authors should put a little more information about mating plugs in other taxonomic groups. Because it is a widely distributed journal, readers in areas other than arachnology may not see interest in the manuscript. I suggest then that the first paragraph of the introduction not go directly to spiders and rather be a very brief summary of mating plug studies in other taxonomic groups.**

Thank you for your suggestions, we have rewritten the introduction accordingly so that the paper is more broadly relevant.

2. **The second problem has to do with the excess of details in the results and the discussion. I understand the need to detail all the structures of the epigynium because the manuscript is through a morphological approach, but the idea is really lost a bit after two full pages of reading the very detailed description of the structures. I suggest that both the results and the discussion be shortened for the reader&#39;s convenience.**

We understand that detailed morphological descriptions are difficult to digest for a reader, but argue that in this case they are needed. We feel that, in order to change the long-standing misconceptions about the structure of the epigynal tracts and to further explore their functions, it is imperative that this paper provides detailed morphological descriptions from various used methods. We have tried our best to improve the readability of the manuscript, and the figure plates can of course be helpful to readers.

The only alternative that might be seen by the reviewer as more appropriate would be to relegate all detailed descriptions (currently under Results) to supplements, and only offer the textual interpretations of the findings within a Results and Discussion section. For the reasons of completeness, and longer durability of this paper once published, we would prefer to retain the current structure of the manuscript.

3. **Finally, although the morphological approach is really good and the detail and comparison between species is really interesting, we know that they are still indirect evidence of the functions of the structures. I really don&#39;t know if the authors performed subsequent experiments with the individuals under laboratory conditions to corroborate the conclusions drawn in the manuscript.**

Our results are in accord with the prediction of the sperm protection hypothesis and thus support the view that formations of SCP and SP are intended as sperm protection mechanisms. Future studies can focus on direct, experimental tests of whether the existence of mating plugs can decrease female remating rates, as we hypothesize.

4. **Abstract
   I understand that a large part of the study was focused on the species D. wulingensis, but I still feel that part of the main results is missing from the comparison that was made with the other species.**

We report these main results of the comparative study: 1) morphology confirms the necessity of a sperm protection mechanism for all entelegyne spiders; 2) the entelegyne spermathecae are divided into four types; 3) due to existence of groove slits between the entrance and exit the SEG type spermathecae of *Diphya wulingensis* requires an additional protection mechanism. In spiders, we conclude, the problem of sperm protection might be resolved in different ways.

**5. Minor fixes
Page 3, line32: I think the authors should provide a little more information about mating plugs both in invertebrates (which is where it is best known) and in vertebrates. Because it is a widely distributed journal, readers from areas other than arachnology may not see interest in the manuscript. I suggest then that the first paragraph of the introduction not go directly to spiders and rather be a very brief summary of mating plug studies in other taxonomic groups.**

Rewritten.

**Page 3, lines 60 and 62, could you please put some references?**

Rewritten.

**Page 5, line 103: missing parentheses to close the sentence.**

Added.

**MATERIALS AND METHODS
Study species
I feel that some biological data is needed on the study species, D. wulingensis, I suggest placing in materials and methods a study species; section on what is known about the species.**

This is included in the paragraph of Line 178.
